# A network-based computational and experimental framework for repurposing compounds toward the treatment of non-alcoholic fatty liver disease

**DOI:** 10.1016/j.isci.2022.103890

**Published:** 2022-02-09

**Authors:** Danae Stella Zareifi, Odysseas Chaliotis, Nafsika Chala, Nikos Meimetis, Maria Sofotasiou, Konstantinos Zeakis, Eirini Pantiora, Antonis Vezakis, George K. Matsopoulos, Georgios Fragulidis, Leonidas G. Alexopoulos

**Affiliations:** 1 School of Mechanical Engineering, National Technical University of Athens, Iroon Polytechneiou 9, Zografou, 15780 Athens, Greece; 2 School of Electrical Engineering, National Technical University of Athens, 15780 Athens, Greece; 3 2nd Department of Surgery, Aretaieio Hospital, University of Athens, School of Medicine, 11528, Athens, Greece; 4 ProtATonce Ltd, Patriarchou Grigoriou & Neapoleos Demokritos Science Park, Building#27, Agia Paraskevi GR15343, Greece

**Keywords:** Pharmaceutical science, Computational bioinformatics, Complex system biology

## Abstract

Non-alcoholic fatty liver disease (NAFLD) is among the most common liver pathologies, however, none approved condition-specific therapy yet exists. The present study introduces a drug repositioning (DR) approach that combines *in vitro* steatosis models with a network-based computational platform, constructed upon genomic data from diseased liver biopsies and compound-treated cell lines, to propose effectively repositioned therapeutic compounds. The introduced *in silico* approach screened 20′000 compounds, while complementary *in vitro* and proteomic assays were developed to test the efficacy of the 46 *in silico* predictions. This approach successfully identified six compounds, including the known anti-steatogenic drugs resveratrol and sirolimus. In short, gallamine triethiotide, diflorasone, fenoterol, and pralidoxime ameliorate steatosis similarly to resveratrol/sirolimus. The implementation holds great potential in reducing screening time in the early drug discovery stages and in delivering promising compounds for *in vivo* testing.

## Highlights

• A computational and experimental drug-screening platform for NAFLD was created
• This framework evaluates *in silico* and validates *in vitro* a great number of compounds
• 20′000 compounds were screened *in silico* and 21 were selected for validation
• Six compounds reversed fully or partially the steatotic phenotype

## Graphical abstract

**Figure:**
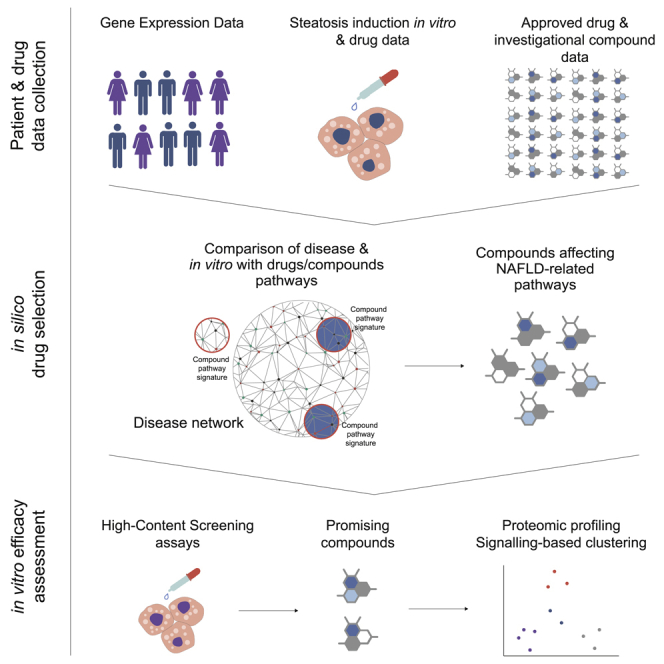

## Introduction

Non-alcoholic fatty liver disease (NAFLD) is becoming one of the most common liver diseases in the world, reaching a global occurrence of almost 25% (Younossi, 2019). NAFLD’|'s pathogenesis is considered involving concurrent genetic, geographic, environmental, and clinical factors that manifest into a spectrum of conditions, ranging from simple steatosis (lipid accumulation in the liver parenchyma) to non-alcoholic steatohepatitis (NASH), fibrosis, and end-stage liver disease. This multi-factorial foundation is associated with several metabolic disorders, including diabetes type-2 and the metabolic syndrome. NAFLD is also an independent factor for cardiovascular disease (CVD) (Rinella et al., 2019).

Current treatment strategies are still limited to provisional changes in diet and lifestyle ("EASL–EASD–EASO Clinical Practice, 2016), while the diagnosis and long-term therapy of chronic patients impose an enormous economic burden (Younossi et al., 2018). Nevertheless, drug discovery is stepping up, with several compounds now reaching clinical trials II and III (i.e. Obeticholic acid, resmetirom). However, none has been yet approved by the European Medicines Agency (EMA) or the US Food Drug Administration (FDA) (Rau and Geier, 2021).

Recent experimental and computational approaches have paved the way to successful drug repositioning (DR) and in turn, to minimize the cost, time, and risk of *de novo* drug discovery. For this purpose, DR identifies and assigns new medical roles to already approved drugs and compounds. Traditionally, experimental DR (eDR) depends on blind experimental screening and inadvertent drug targets (Pushpakom et al., 2019). However, the immense volume of publicly available experimental data has contributed to computational DR (cDR) with an abundance of *in silico* methods. These methods offer a holistic approach by utilizing large-scale drug or disease data to identify and compare either drugs' mode of action, diseases' pathways and networks, or both (Fotis et al., 2018; Jarada et al., 2020; Somolinos et al., 2021). The strategy of comparing pathways and networks that present a hallmark for the disease of interest to those affected by a compound has been successfully used for metabolic disorders (Fotis et al., 2018; Pushpakom et al., 2019)

Thus far, *in silico* and *in vitro* approaches to DR for NAFLD and NASH are promising (Kashyap et al., 2019). On the eDR front, Luo et al. (2018) utilized *in vitro* models and a lengthy high-content screening pipeline to screen the compound library LOPAC1280 (The Library of Pharmacologically Active Compounds, MilliporeSigma, MA, USA), out of which only five were found to demonstrate some repositioning potential (Luo et al., 2018). Alternatively, Sookoian et al. (2019) employed a purely cDR strategy. The authors used publicly available tools and data to map NAFLD and chemical interaction networks, and proposed 149 target genes and compounds interacting with these genes (Sookoian and Pirola, 2019). The lack of an eDR phase imposes the need for additional trials to assess the anti-steatogenic effect, before concluding on the actual repositioning potential. To this end, what remains ambiguous is how effectively cDR can be combined with eDR, leading to repurposed compounds that can be effectively translated into therapeutic interventions. To the best of our knowledge, no robust framework combining cDR and eDR, paired with efficacy testing, for NAFL/NASH exists so far.

This paper introduces an integrated eDR and cDR framework aiming to identify compounds that interfere with mechanisms of liver steatosis toward the amelioration of NAFL. The implemented approach was based on the null hypothesis that if a compound can reverse a pathway that is significantly altered by a disease, it can also reverse the disease phenotype, hence the clinical outcome (Signature Reversion Principle) (Iorio et al., 2015). The first part of the platform consists of the *in* silico selection of repurposing candidates (cDR) based both on gene expression data from clinical human samples and cell-based *in vitro* data. The selected compounds were subsequently validated with *in vitro* assays (eDR), minimizing the overall screening time and increasing hit rate, while broadening the pool of compounds. In detail, gene expression data from patients with NAFL/NASH were obtained from GEO (NCBI) (Edgar et al., 2002) and drug repositories. Second, by combining signature matching and pathway mapping, networks significantly deregulated in NAFL/NASH were identified. These were then compared to networks of approved drugs and investigational compounds, and of compounds used on designed *in vitro* steatosis models, to reveal compounds that could interfere with NAFLD's pathogenesis. Finally, the discovered compounds' efficacy was validated *in vitro* via high-content phenotype screening and proteomic assays.

Out of approximately 20′000 approved and investigational compounds included in the cMap database, cDR screening pinpointed 46 candidates and 21 were moved onto the *in vitro* testing. Out of these, six were found to significantly improve the steatotic phenotype included resveratrol (already been in clinical trials for NAFLD) (Berman et al., 2017; Tejada et al., 2021) and sirolimus (already proven effective *in vitro* and *in vivo*) (Li et al., 2014; Wang, 2010). In addition, proteomic experiments revealed similarities in the compounds' mode of action, thus introducing the possibility for novel therapeutic interventions.

## Results

### Known steatogens induce steatosis *in vitro*

**Figure 1:**
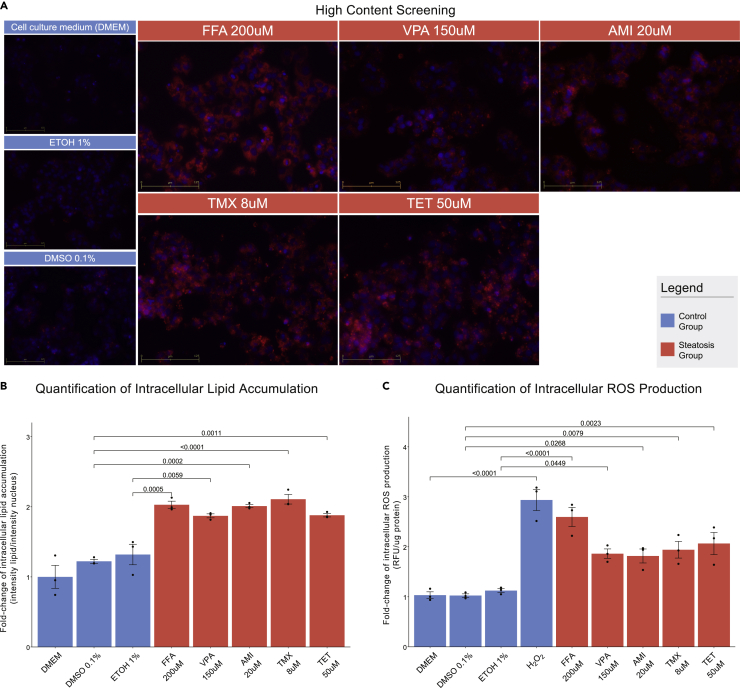
Formation of intracellular lipid droplets and increase of ROS production in HepG2 cells, after treatment with FFA, VPA, AMI, TMX, and TET (A) Intracellular lipid accumulation observed via HCS-based fluorescent microscopy with Nile Red staining. Hoechst33342 was used for staining the nuclei. Images were acquired under 20× optical magnification. (B) Quantification of lipid accumulation via MATLAB-based image analysis. Bars represent the FC of lipid droplet intensity per cell in treated cells over respective controls. (C) FC of intracellular ROS production compared to controls. H_2_O_2_ was used as a positive control. (B, C) Data expressed as mean±SEM of n=3 independent experiments, and the p value is denoted by brackets.

Four hepatic cell lines (HepG2, HuH7, Hep3B, and FOCUS) were treated with a mixture of free fatty acids (FFAs; oleic acid:palmitic acid) and the known steatogenic compounds: valproic acid sodium salt (VPA) (Rodrigues, 2016; Szalowska, 2014; Verrotti, 2009), amiodarone hydrochloride (AMI) (Antherieu, 2011; Szalowska, 2014), tamoxifen citrate (TMX) (Cole, 2010; Yang, 2016; Zhao, 2014), and tetracycline hydrochloride (TET) (Choi, 2015; Szalowska, 2014). Concentrations lower than IC_10_ were used, so to avoid cytotoxic effects, the IC_10_ concentrations were extrapolated from cell-line- and compound-specific dose-viability curves (Figure S1). Intracellular lipid loading was quantified as the intensity of lipid droplets per cell (intensity of nucleus), after Nile Red and Hoechst33342 staining, via MATLAB-based image analysis. Reactive oxygen species (ROS) production was expressed in relative fluorescent units (RFU) per ug of protein. In both, the fold-change (FC) of treated-over-control samples was calculated where the compound's diluent was used as control. The results for the HepG2 cells are shown in Figure 1 for the HuH7, Hep3Β, and FOCUS cells in Figures S2, S3, and S4, respectively. Briefly, all treatments led to a significant increase in both intracellular lipid droplet formation and ROS production across all the cell lines, except for TMX on HuH7 cells, thus recapitulating the steatotic phenotype. The steatogens' targets and pathways affected were identified through DrugBank and MSigDB databases forming the “steatogenic compounds' pathways”.

### Significantly deregulated pathways in NAFL and NASH via GLS and GSA analysis on clinical data

**Figure 2:**
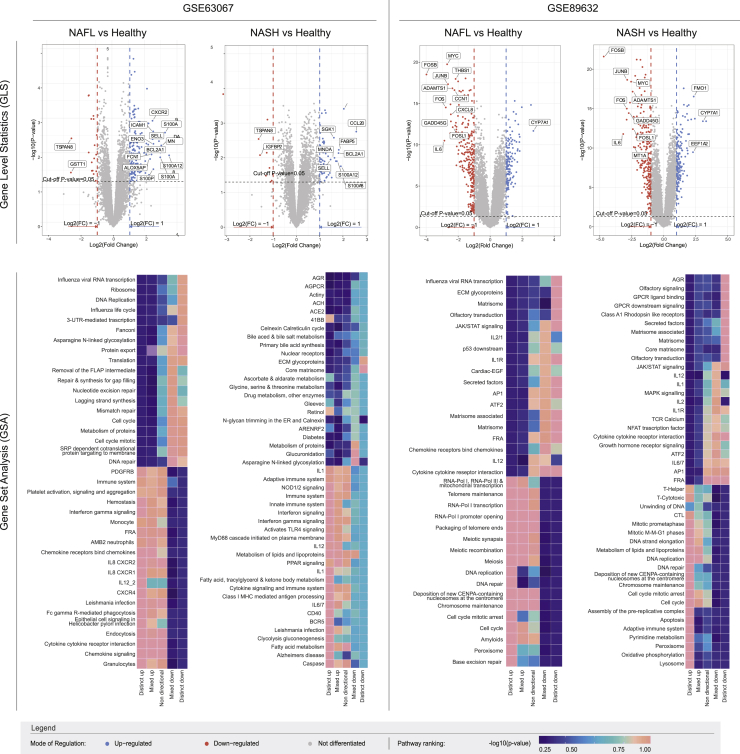
Gene levels statistics (GLS) and gene set analysis (GSA) from microarray gene expression datasets denoting the 15 most statistically significant differentially expressed genes and the differentially altered pathways The degree of differential gene expression was calculated for each sample as the logarithm of the fold-change (FC) of expression values in the diseased stages (test) over the healthy state (control). A student's t-test was used for statistical evaluation. For graphically representing these data, log_2_ FC and p value for each gene was plotted in volcano plots. Each point on the plot corresponds to a gene, while the y axis represents the negative decimal log of p−value and the x axis represents log_2_ FC. The greater the difference of the gene on the vertical axis compared to the control group, the more statistically significant the differential expression, and the farther from zero on the horizontal axis, the greater the intensity. $|{\log\;}_{2}\;FC|>1$ and p-value ≤ 0.05 are used as the limits for the differential expression of a gene. Results of GLS are presented in heatmaps. Νine different statistical methods were utilized to identify the prevalent expression trend within a pathway. Based on their prevalent trend and p value, pathways were classified and ranked into five groups, namely “distinct up”, “mixed up”, “non-directional”, “mixed down”, and “distinct down” according to their given “-value” Each column represents one of the clusters. Each row corresponds to a pathway. Color scale denotes statistical significance (-log10(p-value)).

For each dataset selected (Table 1), gene-level statistics (GLS) via the limma package (Ritchie et al., 2015) revealed the statistically significant differentially expressed genes (DE-Gs) in the “NAFL” and “NASH” states against the “Healthy” control state. The results were input for the gene set analysis PIANO package (Väremo et al., 2013). The analysis produced significantly affected pathways in “NAFL” and “NASH” and divided them into five classes, according to their expression trend (Figure 2). The significantly affected pathways formed the group of “clinical data pathways”.

**Table 1:** Datasets of microarray gene expression profiling from patients with biopsy-proven NAFLD/NASH and healthy individuals obtained from GEO (NCBI)

GEO series accession	Contributors	Number of control samples	Number of diseased samples	Pathological phenotype of NAFL
GSE63067	Frades I et al. (Frades et al., 2015)	7	11	NASH, Steatosis
GSE89632	Arendt et al. (Arendt et al., 2015)	24	39	NASH, Steatosis

### Identification of pathways affected by known steatogenic compounds

The known steatogens, used *in vitro*, were integrated into the platform in a two-step manner. Firstly, they were input to the DrugBank (Wishart et al., 2006) and the GSEA-MSigDB (Liberzon et al., 2015) databases to identify their targets and the pathways affected, forming the “steatogenic compounds” pathways group. The intersection between this group and that of the “clinical data pathways” is shown in Table S1. The known steatogens were introduced to the Connectivity Map (cMap) (Broad Institute) (Lamb, 2006; Subramanian et al., 2017) to identify FDA-approved compounds with similar or opposite gene signature (repositioned compounds). The DrugBank and MSigDB databases were again used to identify the pathways that these repositioned compounds affect, to form a group of “DR compounds' pathways” (Tables S2–S10).

### Eleven (11) pathways-to-target and 46 compounds were identified based on network similarity between the clinical and *in vitro* data

**Figure 3:**
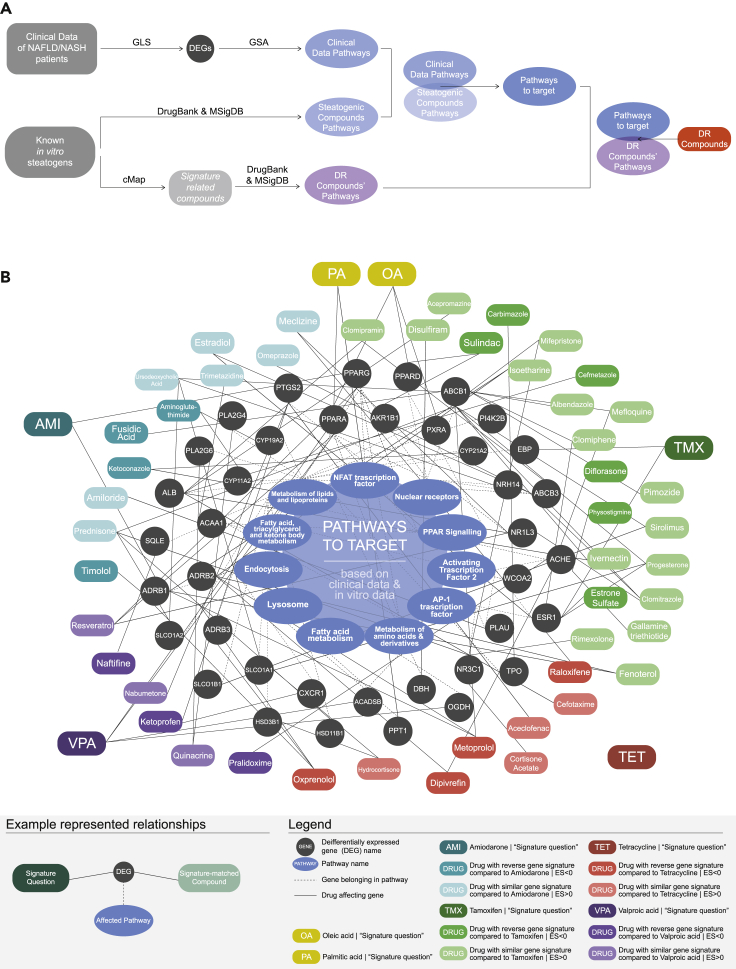
Network representation of the identification process and resulting repurposed compounds, as proposed by the DR platform (A) Schematic representation of the repurposed compounds identification process. (B) The target pathways depicted in blue in the center of the graph belong at the intersection of the “Clinical Data Pathway Group” and “Steatogenic Compounds Pathway Group”. The differentially expressed genes (DE-Gs) and the pathways affected by each compound are illustrated in gray circles. Each steatosis-inducing compound was used as a “signature question” to cMap. The cMap tool compares two-sample distributions using the Kolmogorov-Smirnov (K-S) statistical test and calculates an Enrichment Score that takes values in the interval [-1,1]. ES > 0 signifies that two drugs present similar gene signature, while ES < 0 means that two drugs have reverse gene signatures. The steatosis-inducing compounds are depicted in rectangles of different colors. Every steatosis-inducing compound drugs with ES > 0 or ES < 0 is illustrated with rectangles of the same color scale. Lines connect each compound and drug with their target genes and the pathways they affect.

The comparison between the “clinical data pathways” and the pathways targeted by the steatogenic compounds used *in vitro*, or the “steatogenic compounds' pathways”, revealed 11 significant deregulated pathways in NAFL/NASH, hence the pathways-to-target (Figure 3). Namely, these pathways were the nuclear receptors pathway (BIOCARTA, M16393), the endocytosis pathway (KEGG, ko04144), the PPAR-signaling pathway (KEGG, HSA-03320), the fatty acid metabolism pathway (KEGG, map01212), the activator protein-1 pathway (PID, M167), the activating transcription factor 2 pathway (PID, M166), the NFAT transcription factor pathway (PID, M60), the fatty acid triacylglycerol and ketone body metabolism pathway (REACTOME, R-HSA-188467), the metabolism of amino acids and derivatives pathway (REACTOME, R-HSA-71291), and the metabolism of lipids and lipoproteins pathway (REACTOME, R-HSA-556833) (Fabregat et al., 2018; Jassal et al., 2020; Kanehisa and Goto, 2000; Mootha et al., 2003; Nishimura, 2004; Schaefer et al., 2009). For both datasets, volcano plots, provided in Figure S5, depict the DE-Gs in the identified pathways-to-target.

Approximately 20′000 approved and experimental compounds were screened *in silico* for their ability to affect the aforementioned pathways. Forty-six (46) compounds were found to interfere with both the “DR compounds' pathways” and the identified pathways-to-target and were therefore proposed as promising for the treatment of hepatic steatosis. Figure 3B illustrates the repositioned compounds, as well as the genes and pathways affected by these compounds.

### Experimental validation of candidate compounds resulting from *in silico* selection revealed six with anti-steatotic effect

The 46 candidate compounds were reviewed on the ToxDB and the LiverTox databases. Twenty-five (25) were documented to be hepatotoxic or to induce steatosis *in vitro* or *in vivo* and were, thus, eliminated. The remaining 21 moved onto screening to investigate their capacity to reduce steatosis and oxidative stress *in vitro*. Those were acepromazine, cefmetazole, clomifene, diflorasone, estradiol, estrone sulfate, fenoterol, fusidic acid, gallamine triethiotide, ivermectin, mefloquine, naftifine, pimozide, pralidoxime, quinacrine, raloxifene, resveratrol, rimexolone, sirolimus (or Rapamycin), and timolol.

**Figure 4:**
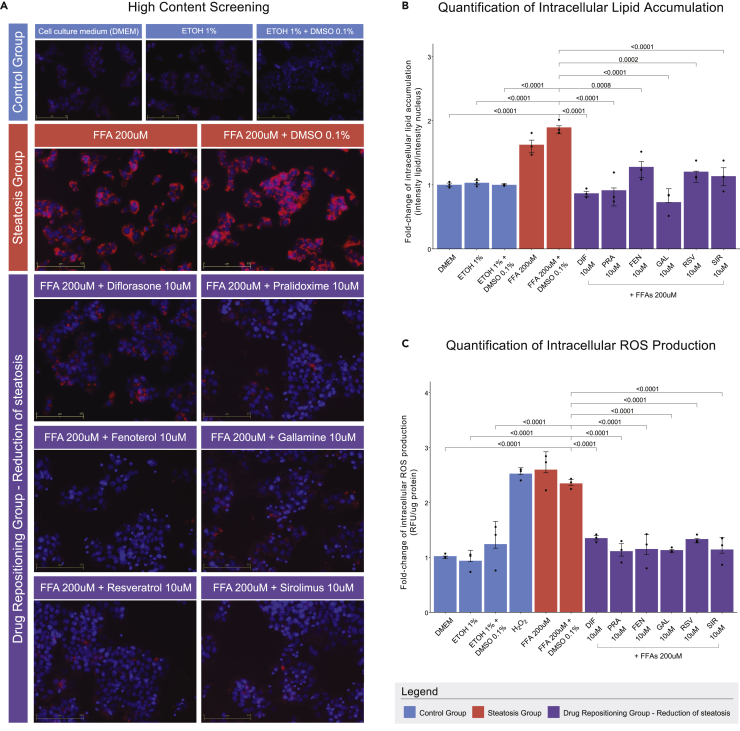
Reduction of intracellular lipid accumulation and ROS production in HepG2 cells, after treatment with the repositioned compounds (A) Intracellular lipid accumulation observed via HCS-based fluorescent microscopy with Nile Red staining. Hoechst33342 was used for staining the nuclei. Images were acquired under 20× optical magnification. (B) Quantification of lipid accumulation via MATLAB-based image analysis. Bars represent the FC of lipid droplet intensity per cell in treated cells over respective controls. (C) FC of intracellular ROS production compared to controls. H_2_O_2_ was used as a positive control. (B, C) Data expressed as mean±SEM of n=3 independent experiments, and the p value is denoted by brackets.

High-content screening of the 21 compounds was performed on HepG2 cells. Verified compounds would demonstrate the capacity to reduce intracellular lipid accumulation and ROS production in steatotic cell cultures. Pimozide (PIM), clomifene (CLO), and mefloquine (MEF) led to a significant increase (p value<0.05) in the cells' intracellular lipid loading and ROS production (Figure S6). Conversely, HepG2 cells, co-treated with FFAs 200uM and either sirolimus (SIR), resveratrol (RES), diflorasone (DIF), fenoterol (FEN), pralidoxime (PRA), or gallamine triethiotide (GAL) were found to effectively ameliorate the steatotic phenotype at 10uM, while none reduced cell viability below 80%. In detail, all six co-treatments led to a significant (p value<0.05) reduction in lipid loading and oxidative stress, when compared to the FFA-treated control (Figure 4). Equally important, lipid loading and oxidative stress were not significantly increased in cells treated solely with the six DR compounds (Figure S7).

### Signaling-based clustering reveals the anti-steatotic efficacy of repositioned compounds

**Figure 5:**
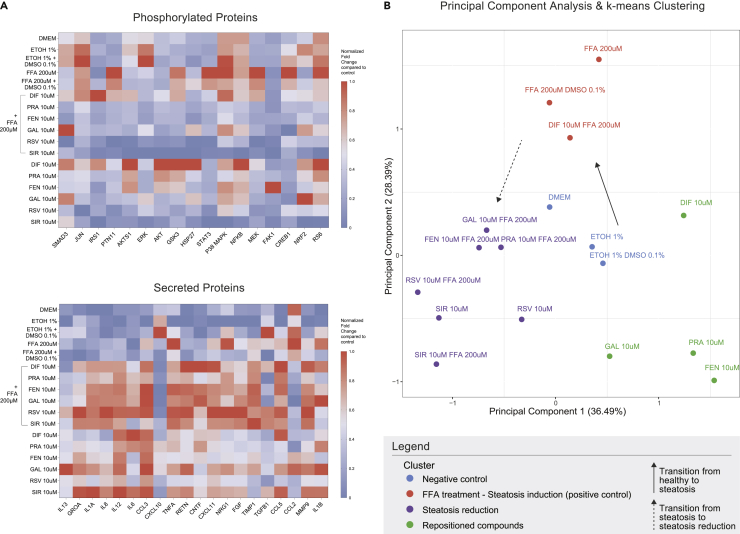
Proteomic profiling of the effect of the compounds (A) Heatmap of the normalized fold change of phosphorylated proteins and secreted cytokines compared to controls. Each column corresponds to a protein and each row to a cell treatment. Data in each column were fraction-normalized to [0–1] scale for better representation. The color scale represents the value of normalized fold change compared to the control. See also Figures S9–S20. (B) PCA and k-means clustering was performed on proteomic, lipid accumulation, and intracellular ROS production data. The axes denote the first two principal components and the percentage of variability they contribute. k-means was used to deduce the clusters of the resulting profile of the compound treatment. The four different clusters formed are denoted with different colors and were named after the majority of treatments included. The solid arrow represents the transition from control to steatosis state, whereas the dashed arrow represents the transition from steatosis to an amelioration of steatosis state.

The measurements on the phospho-protein and cytokine-release levels are summarized in Figures 5A and S9–S20. All compounds were investigated for their shared motifs at the protein signaling level. Principal component analysis (PCA) on the whole of the experimental data, followed by k-means clustering (Figure 5B), revealed 4 clusters distinguished by their anti-steatogenic mode of action. All of the negative controls (DMEM, ETOH 1%, ETOH 1%, and DMSO 0.1%) were appropriately clustered together (“negative control” cluster). All FFA-treated samples (FFA 200uM, FFA 200uM, and DMSO 0.1%) were grouped into a second, “steatosis” cluster. Sole administration of DIF, GAL, PRA, and FEN fell into a third distinct cluster. Importantly, all the rest of the DR co-treatments, except for DIF, were grouped with SIR + FFAs, as well as RSV + FFAs; the DR compounds that have been already proven effective *in vitro* or *in vivo* for the treatment of NAFL. The DIF + FFAs co-treatment failed to cluster with the DR candidates and was clustered into the steatosis cluster.

## Discussion

In this work, a compound-selection framework for NAFL/NASH has been developed to capitalize on the effective combination of *in silico* speed and *in vitro* efficacy validation. Applied to the set of 20′000 compounds comprising the cMap database, this approach successfully selected two compounds with documented efficacy and revealed 19 new and two known compounds with anti-steatogenic potential. Several of the novel predictions were experimentally confirmed using high-throughput bioassays and *in vitro* steatosis models, and demonstrated the framework's efficiency in discovering anti-steatogenic compounds for the amelioration of NAFL.

In contrast to other cDR methods, the proposed *in silico* selection of compounds was integrated with existing *in vitro* models, hence directed toward compounds that could be validated *in vitro*. For that, the differential gene expression between healthy and NAFL/NASH samples was compared to the gene expression profile of compounds used for *in vitro* steatosis models. Just as important, on the eDR front, the development of *in vitro* models and their incorporation in the *in silico* analysis allowed for the development of a high-throughput drug-screening setup for the validation and efficacy testing of the computational predictions, reaching a very high *in vitro* screening hit rate of 28.5% (out of the 21 screened compounds, six showed positive anti-steatotic results).

For the experimental drug-screening pipeline, four hepatic cell lines were treated with known *in vitro* and *in vivo* steatogens to create *in vitro* steatosis models and successfully recapitulated the steatotic phenotype. Consistent with previous *in vitro* studies (15–23), all the compounds were found to increase intracellular lipid accumulation and oxidative stress across all cell lines except HuH7.

Pathways affected by 20′000 FDA-approved and investigational compounds, included in the cMap database, were then compared to the pathways identified *in silico* from clinical and *in vitro* data. Therefore, the cDR platform identified pathways significantly affected both in patients with NAFL/NASH and in the *in vitro* models and suggested 46 compounds as capable of interfering with the disease's pathways. Out of these, 25 were eliminated from further analysis as known hepatotoxic, while the remaining were examined for their capacity in reducing the intracellular lipid accumulation and oxidative stress with a high-content screening setup.

Six (6) compounds (sirolimus, resveratrol, gallamine triethiotide, fenoterol, pralidoxime, and diflorasone) out of the 21 screened *in vitro* succeeded in effectively ameliorating the steatotic phenotype *in vitro* (Figure S8). In all co-treatments with FFAs and the identified compounds, cells showed reduced oxidative stress and lipid loading, not at the expense of cell viability. Specifically, on sirolimus and resveratrol, the platform succeeded in confirming the documented *in vitro* and *in vivo* anti-steatogenic effects (Ali et al., 2015; Berman et al., 2017; Bujanda et al., 2008; Charytoniuk et al., 2017; Wang et al., 2014; Wang, 2010). As the *in silico* analysis was non-directional, it also identified compounds that induce or deteriorate steatosis *in vitro*. In detail, pimozide, clomifene, and mefloquine underwent a verification process but were found to aggravate steatosis *in vitro*. Nonetheless, as all tests were performed at a compound concentration of 10uM; further investigation is required toward an optimal, compound-specific concentration.

Proteomic profiling with a diverse panel of phosphorylated proteins and secreted cytokines was performed to deduce the compounds' signaling motifs. PCA and k-means clustering led to the formation of four clusters, named “Negative control”, “FFA induction”, “Steatosis reduction”, and “Repositioned compounds” after the majority of treatments that comprised each of them. Firstly, this clustering revealed a distinct difference between all of the negative control samples (DMEM, etOH 1%, etOH 1%, & DMSO 0.1%) and the FFA-treated samples, underlining that the variables of the analysis were able to distinguish between the healthy and the steatotic phenotype, based on their respective signaling mode of action. Most importantly, all co-treatments with the repositioned compounds, except for DIF, were found in the same cluster with the SIR + FFAs and RSV + FFAs, thus highlighting GAL's, FEN's, and PRA's potential in ameliorating *in vitro* steatosis at the pathway level. Regarding DIF, when used in co-treatment with FFAs, it is clustered together with the FFA-treated controls, a finding that comes into conflict with DIF's observed capacity to lower lipid loading and oxidative stress. DIF's variability in the proteomic measurements is the primary reason for the misclassification, suggesting either a different mode of action than the one captured with the used proteomic panel, or a dose that was insufficient to reverse steatosis at the pathway level. The fourth cluster consisted of samples treated with the repositioned compounds alone, except for RSV and SIR that cluster with the anti-steatosis group, suggesting a high impact of RSV's and SIR's mode of action on this cluster. This impact can be attributed to the effect of these drugs on the proteins selected in the multiplex panel.

Besides its limitations, this paper is contributing to the integration of an *in silico* and an *in vitro* screening pipeline for DR and acknowledges that additional experiments are needed to decipher the compounds' mode of action and anti-steatogenic effect.

In conclusion, this framework allows for the evaluation of a great number of compounds, at the early stages of drug discovery, by combining the large compound-examination capacity, offered by the *in silico* models, with the rigor of the *in vitro* validation. The attempted implementation actively saves up screening time, as several candidates were eliminated *in silico*, long before their verification *in vitro*. Albeit the gap between *in vitro* validation and clinical efficacy, the proposed framework enables the exploration of the large chemical space and delivers promising compounds for subsequent *in vivo* efficacy studies. This strategy provides a basis for evaluating the repositioning potential of widely used drugs, even beyond NAFL, and, as such, the illustrated framework holds significant potential in assisting the treatment of several diseases.

### Limitations of the study

The present study comes with certain limitations. The use of 2D *in vitro* models, although sufficient for screening, cannot recapitulate the complexity of human NAFLD pathophysiology (Müller and Sturla, 2019). Limitations imposed by the simplified 2-dimensional steatosis models can be surpassed with additional *in vivo* and *in vitro* studies in relevant 3D liver models or other animal NAFLD models.

## STAR★Methods

### Key resources table

**Table:** 

REAGENT or RESOURCE	SOURCE	IDENTIFIER
**Antibodies**		

Cytokines bead mix	Proatonce Ltd.	PR-CU060-BM-20
Cytokines Detection mix	Proatonce Ltd.	PR-CU060-DM-20
Phosphorylated proteins Coupled bead mix	Proatonce Ltd.	PR-CU060-BM-17
Phosphorylated proteins Detection mix	Proatonce Ltd.	PR-CU060-DM-17
SAPE	Proatonce Ltd.	PR-SAPE

**Chemicals, peptides, and recombinant proteins**		

Oleic acid	Cayman Chemical	90260
Palmitic acid	Cayman Chemical	10006627
Valproic acid sodium salt	Cayman Chemical	13033
Tetracycline hydrochloride	Cayman Chemical	14328
Amiodarine hydrochloride	Cayman Chemical	15213
Tamoxifen citrate	Cayman Chemical	11629
Timolol maleate	Cayman Chemical	13974
Fenoterol hydrochloride	Cayman Chemical	21293
Naftifine hydrochloride	Cayman Chemical	19234
Pimozide	Cayman Chemical	16222
Acepromazine maleate	MilliporeSigma	LOPAC®1280, LO4200-1EA
Cefmetazole sodium salt	MilliporeSigma	LOPAC®1280, LO4200-1EA
Clomiphene citrate	TargetMol	T1193
Diflorasone diacetate	Cayman Chemical	23808
Estradiol	MilliporeSigma	LOPAC®1280, LO4200-1EA
Estrone sulfate	MilliporeSigma	LOPAC®1280, LO4200-1EA
Fusidic acid sodium salt	MilliporeSigma	LOPAC®1280, LO4200-1EA
Gallamine triethiotide	MP Biomedicals	0521278880
Ivermectin	MilliporeSigma	LOPAC®1280, LO4200-1EA
Mefloquine hydrochloride	Cayman Chemical	23665
Pralidoxime chloride	TargetMol	T1111
Quinacrine dihydrochloride	MilliporeSigma	LOPAC®1280, LO4200-1EA
Raloxifene hydrochloride	MilliporeSigma	LOPAC®1280, LO4200-1EA
Resveratrol	TargetMol	T1558
Sirolimus (rapamycin)	MilliporeSigma	37095
Bovine Serum Albumin	MilliporeSigma	A7638
Dulbecco′s Modified Eagle′s Medium (DMEM)	Biosera	LM-D1113
Fetal Bovine Serum (FBS)	Biosera	FB-1001
Penicilin-Streptomycin solution	Biosera	XC-A4122
Ethanol	MilliporeSigma	1009835000
DMSO	MP Biomedicals	11DMSO0001
Hoechst 33342	Thermo Fisher Scientific	H3570
Nile Red	Thermo Fisher Scientific	N1142
CM-H_2_DCFDA	Thermo Fisher Scientific	C6827
Resazurin sodium salt	MilliporeSigma	R7017
PMSF (Phenylmethylsulfonyl fluoride)	MilliporeSigma	P7626
Protease Inhibitors	Proatonce Ltd.	PR-PI
Lysis Buffer	Proatonce Ltd.	PR-LYSB

**Critical commercial assays**		

Pierce™ BCA Protein Assay Kit	Thermo Fisher Scientific	23227

**Deposited data**		

Expression data from human non-alcoholic fatty liver disease stages	https://www.ncbi.nlm.nih.gov/geo/query/acc.cgi	GSE63067
Genome-wide analysis of hepatic gene expression in patients with non-alcoholic fatty liver disease and in healthy donors in relation to hepatic fatty acid composition and other nutritional factors	https://www.ncbi.nlm.nih.gov/geo/query/acc.cgi	GSE89632
L1000 Connectivity Map perturbational profiles from Broad Institute LINCS Center for Transcriptomics LINCS Pilot PHASE I	https://www.ncbi.nlm.nih.gov/geo/query/acc.cgi?acc=GSE92742	GSE92742
Molecular Signatures Database	http://www.gsea-msigdb.org/gsea/msigdb/index.jsp	MSigDB
Toxicogenomics Database	http://toxdb.molgen.mpg.de/	ToxDB
LiverTox	https://www.ncbi.nlm.nih.gov/books/NBK547852/	LiverTox

**Experimental models: cell lines**		

HepG2	ATCC®	HB-8065
HuH7	A kind gift from J. Wands (Brown University)	
Hep3B	ATCC®	HB-8064
FOCUS	A kind gift from J. Wands (Brown University)	

**Software and algorithms**		

R Programming language v3.4	Bell Laboratories	https://www.r-project.org/
MATLAB R2017A	Mathworks	https://www.mathworks.com/products/matlab.html
ConnectivityMap	Broad Institute	L1000 (https://clue.io/cmap#access)
Image analysis code	https://github.com/BioSysLab/NAFLD_computational_analysis	• https://github.com/BioSysLab/NAFLD_computational_analysis/blob/main/final_program_counting_cores_and_lipids.m • https://github.com/BioSysLab/NAFLD_computational_analysis/blob/main/pre_process1.m • https://github.com/BioSysLab/NAFLD_computational_analysis/blob/main/createCirclesMask.m
Computational drug repositioning code	https://github.com/BioSysLab/NAFLD_computational_analysis	• https://github.com/BioSysLab/NAFLD_computational_analysis/blob/main/gsea_path_analysis_pipeline.R •https://github.com/BioSysLab/NAFLD_computational_analysis/blob/main/gene_expression_fig2.R
Statistical analysis code	https://github.com/BioSysLab/NAFLD_computational_analysis	• https://github.com/BioSysLab/NAFLD_computational_analysis/blob/main/tukey_final_imaging.R • https://github.com/BioSysLab/NAFLD_computational_analysis/blob/main/tukey_final_ROS_DRsteat.R • https://github.com/BioSysLab/NAFLD_computational_analysis/blob/main/proteomics.R • https://github.com/BioSysLab/NAFLD_computational_analysis/blob/main/PCA.R

**Other**		

Human liver biopsy of different phases from control to NASH	https://www.ncbi.nlm.nih.gov/geo/query/acc.cgi?acc=GSE48452	GSE48452
Expression data from human non-alcoholic fatty liver disease stages	https://www.ncbi.nlm.nih.gov/geo/query/acc.cgi	GSE63067
Genome-wide analysis of long noncoding RNA expression profile in non-alcoholic fatty liver disease	https://www.ncbi.nlm.nih.gov/geo/query/acc.cgi	GSE72756
Genome-wide analysis of hepatic gene expression in patients with non-alcoholic fatty liver disease and in healthy donors in relation to hepatic fatty acid composition and other nutritional factors	https://www.ncbi.nlm.nih.gov/geo/query/acc.cgi	GSE89632
L1000 Connectivity Map perturbational profiles from Broad Institute LINCS Center for Transcriptomics LINCS Pilot PHASE I	https://www.ncbi.nlm.nih.gov/geo/query/acc.cgi?acc=GSE92742	GSE92742
Molecular Signatures Database	http://www.gsea-msigdb.org/gsea/msigdb/index.jsp	MSigDB
Toxicogenomics Database	http://toxdb.molgen.mpg.de/	ToxDB
LiverTox	https://www.ncbi.nlm.nih.gov/books/NBK547852/	LiverTox

### Resource availability

#### Lead contact

Further information and requests for resources and reagents should be directed to and will be fulfilled by the lead contact, Leonidas G. Alexopoulos (leo@mail.ntua.gr).

#### Materials availability

This study did not generate new unique reagents.

### Experimental models details

Four hepatic cell lines, HUH7, HepG2, Hep3B and FOCUS, were cultured in Dulbecco′s Modified Eagle′s High Glucose Medium (DMEM) (Biosera, Nuaille, France) supplemented with 10% v/v Fetal Bovine Serum (FBS) (Biosera, Nuaille, France) and 1% v/v Penicillin-Streptomycin solution (Penicillin: 10'000 units/mL; Streptomycin: 10'000 ug/mL) (Biosera, Nuaille, France), at a 37°C, 5% CO_2_ humidified incubator. For drug treatment, compounds were diluted in serum-free medium without phenol red (Thermo Fisher Scientific, MA, USA) at either 0.1%v.v DMSO or 1% v/v etOH.

### Methods details

#### *In vitro* steatosis induction

Before treatment, the cells were seeded onto black flat-bottomed 96-well plates for 24 h at the corresponding densities: HUH7: 15'000 cells/well, HepG2: 20'000 cells/well, Hep3B: 15'000 cells/well, FOCUS: 10'000 cells/well in serum-free medium.

Cells were treated for 24h in serum-free medium with known steatogenic compounds. Namely, free fatty acids (FFAs; oleic acid:palmitic acid) (Cayman Chemical, MI, USA), Valproic acid sodium salt (VPA) (Cayman Chemical, MI, USA), Amiodarone hydrochloride (AMI) (Cayman Chemical, MI, USA), Tamoxifen citrate (TMX) (Cayman Chemical, MI, USA) and Tetracycline hydrochloride (TET) (Cayman Chemical, MI, USA) were evaluated as known steatogens. Oleic acid (OA), palmitic acid (PA), and VPA were diluted in 100% ethanol (etOH) at 1% v/v final (% in cell culture medium) concentration. TMX, AMI and TET were diluted in DMSO at 0.1% v/v final concentration. Exogenous FFAs (molar ratio OA:PA = 2:1) were conjugated with Bovine Serum Albumin (BSA) (MilliporeSigma, MA, USA) at a molar ratio of FFAs:BSA = 4:1.

#### Verification of lipid droplet accumulation via high-content screening (HCS)

Lipid droplets were fluorescently stained with Nile Red (Thermo Fisher Scientific, MA, USA) and cell nuclei were counterstained with Hoechst 33342 (Thermo Fisher Scientific, MA, USA). The culture medium was first aspirated and the cells were rinsed three times with Phosphate-buffered Saline (PBS) buffer (Biosera, Nuaille, France). Nile Red and Hoechst 33342 were diluted in Phenol-Red-free culture medium at 4ug/mL and 5ug/mL final concentrations respectively. Thirty (30) uL of the imaging medium were added into each well and plates were then incubated for 45min at 37°C. Images were acquired automatically using JuLI™ Stage Real-Time CHR (NanoEnek, Seoul, Korea) with a 20x objective lens of a high-sensitivity monochrome CCD camera (Sony sensor 2/3") at room temperature.

#### Image analysis

Lipid accumulation was computationally quantified with image analysis, given the images obtained with HCS. Five images were acquired per well and all experiments were performed in technical and experimental replicates, hence a minimum of 50 images were analysed per treatment. An image analysis pipeline was created in MATLAB (v.2018a, Mathworks, USA). In short, multi-channel images were divided into the corresponding channels and converted into a binary format through a number of filters. Object sharpening and background elimination led to the identification and labelling of cell boundaries, nuclei and lipid droplets (Methods S1). For the output, the number of nuclei and the number of lipid droplets and droplet area were extracted.

#### Quantification of oxidative stress

Reactive oxygen species (ROS) were quantified with the CM-H_2_DCFDA fluorescent substrate (Thermo Fisher Scientific, MA, USA). Cells, treated with 100uM of H_2_O_2_ for 30 min, were used as a positive control. CM-H_2_DCFDA was diluted in Phenol-Red-free and sodium-pyruvate-free culture medium at 10uM final concentration (Thermo Fisher Scientific, MA, USA). Thirty (30) uL of the staining solution were added per well. The plates were then incubated for 45 min at 37°C. After incubation, fluorescence was measured at Ex495 nm/Em520 nm and normalized per ug of protein. The total protein concentration was deduced using BCA^TM^ assay (Thermo Fisher Scientific, MA, USA) for each sample. CM-H_2_DCFDA fluorescence and BCA absorbance were quantified with the Varioskan^TM^ LUX multi-mode microplate reader (Thermo Fisher Scientific, MA, USA).

#### Resazurin reduction cell viability assay

Cell viability was quantified with Resazurin (MilliporeSigma, MA, USA). The Resazurin solution was added to the cell culture medium at a final concentration of 10ug/mL. The plates were then incubated for 2 hours at 37°C. Fluorescence was measured at Ex560 nm/Em590 nm, in Relative Fluorescent Units (RFU), with the Varioskan^TM^ LUX multi-mode microplate reader (Thermo Fisher Scientific, MA, USA). The sample’s viability was approximated as a percentage of treated over untreated cells. The experimental data were then fitted onto a 4-parameter logistic regression model in GraphPad Prism 9.0. ΙC_10_ values were extrapolated.

#### Identification of “clinical data pathways”

Datasets of microarray gene expression profiling, derived from biopsied NAFLD/NASH patients and healthy individuals, were obtained from the GEO (NCBI) database (Edgar et al., 2002). To assure robustness, only datasets with a sufficient number of samples and differentially expressed genes (DEGs) were selected, based on their degree of differential expression. The degree of differential expression was calculated for each sample as the logarithm of the fold-change (FC) of expression values in the diseased condition (test) over the healthy condition (control). A student's t-test was used for statistical evaluation. Next, each of the selected datasets was subjected to gene-level statistics (GLS), followed by pathway analysis (Gene Set Analysis; GSA). For the pathway analysis of clinical and compounds' data BIOCARTA (Nishimura, 2004), Protein Interaction Database (PID) (Schaefer et al., 2009), KEGG (Kanehisa and Goto, 2000; Mootha et al., 2003) and REACTOME (Fabregat et al., 2018; Jassal et al., 2020) databases were used as a knowledgebase.

GLS calculated the degree of differential expression for each gene via R Bioconductor's LIMMA (Linear Models for Micro-array and RNA-seq Data) package (Ritchie et al., 2015). For each dataset, samples were divided into three clusters according to their health status (Healthy, NAFL or NASH). For every gene in a dataset, a linear model was generated in order to quantify the degree of differential expression. To eliminate false positives, hierarchical models were used to describe the coefficients of variation and to express variation as a function of the genes. The "Healthy" cluster was compared to both "NAFL" and "NASH". Finally, through the empirical Bayes methods, the B-value hyperparameter and a moderated t-statistic were calculated.

GSA (or pathway analysis) identifies significantly affected pathways. The analysis was performed with R Bioconductor's PIANO (Platform for Integrative Analysis of Omics Data) package under a functional class-scoring method (FCS) (Mathur et al., 2018; Väremo et al., 2013). Genesets, provided by MSigDB, and GLS output underwent pathway analysis. For that, nine different statistical methods were utilized to identify the prevalent expression trend within a pathway (Methods S2). Pathways were classified and ranked into five groups based on the gene expression trend and p-value within a pathway, namely "distinct up", "mixed up", "non-directional", "mixed down" and "distinct down" according to their given p-value (Methods S3). Those with low *p*-values were considered to be the most significant in terms of differential expression. Taken together, they formed a group of "clinical data pathways". Notably, both NAFLD and NASH states were evaluated to establish the pathways involved in the critical progression from simple steatosis to steatohepatitis.

#### Identification of "steatogenic compounds' pathways"

The compounds' used to induce steatosis *in vitro* were interrogated against the DrugBank (Wishart et al., 2006) and MSigDB (Liberzon et al., 2015) databases. The compounds were used as input to DrugBank, from which, target genes and genes of relative enzymes, transporter, and carrier proteins were pooled. This set of genes was then fed to MSigDB to retrieve the pathways they belong to. The output forms the "steatogenic compounds' pathways" group.

#### Identification of "DR compounds' pathways"

The known steatogens were input to the Connectivity Map (cMap) database (Broad Institute) (Lamb, 2006; Subramanian et al., 2017). cMap compares differences in gene expression levels, termed "signatures", between a disease, genetic perturbation or treatment with a small molecule ("signature questions"), to all perturbational signatures available. The similarity comparisons are evaluated through the Kolmogorov-Smirnov (K-S) test (Kim and Volsky, 2005), where an Enrichment Score (ES) is calculated within the [-1,1] interval. Positive values of the ES correspond to similar signatures, while negative correspond to opposite signatures.

For each of the known steatogens, posed as the input "signature question", cMap returned a table of bioactive compounds was produced along with their ES scores. Only compounds with significantly (p-value<0.05) similar (ES>0) or opposite (ES<0) mode of action were selected. Out of those, known hepatotoxic and steatogenic compounds were reviewed on the ToxDB (Hardt et al., 2016) and LiverTox (Hoofnagle et al., 2013) databases to be excluded from further analysis. Each of the selected compounds was then introduced to DrugBank to identify relative target genes that were then input to MSigDB. MSigDB returned those pathways containing the target genes each compound affects. Those pathways, taken together, formed a group of "DR compounds' pathways".

#### Comparison among pathway groups for identification of pathways-to-target

The analysis so far contributed 3 groups of pathways: the “clinical data pathways”, the "steatogenic compounds' pathways", and the "DR compounds' pathways". The overlap between the “clinical data pathways” and the "steatogenic compounds' pathways" consists of pathways involved in the induction of steatosis that can be recapitulated *in vitro*. This set of overlapping pathways also coincides with a portion of the "DR compounds' pathways", extracted from cMap. It was hypothesized that the pathways-to-target belong to the intersection of those groups of pathways, as, on one hand, interfere with *in vitro* steatosis, and on the other hand, demonstrate similar or opposite modes of action to the known *in vitro* steatogens. The compounds to be tested were traced back to these pathways-to-target in the backward fashion, based on the pathway-target identification approaches described in traditional network-based frameworks (Fotis et al., 2018).

#### Validation of the *in silico* predictions with High-Content Screening (HCS)

An HCS pipeline was devised to verify the *in vitro* effects of the proposed DR compounds. HepG2 cells were seeded in black, flat-bottomed, 384-well plates at 5'000/well density and treated with FFAs at 200uM. Control samples were treated only with the candidate DR compounds at 10uM, while the rest were treated with a co-treatment of FFAs and DR compounds to evaluate the anti-steatogenic potential. All treatments were applied for 24h before HCS. Intracellular lipid content and ROS production were quantified according to the assays described in §2.1.2, §2.1.3 and §2.1.4.

#### Protein isolation

Total protein isolation protocols were followed for phospho-proteomic measurements. The cells were seeded on flat-bottomed, 96-well plates at their aforementioned densities. After 24h, they were co-treated with FFAs at 200uM and the DR compounds at 10uM for another 24h before lysis. The lysis buffer (ProtAtOnce Ltd, Athens, Greece) was supplemented with a protease/phosphatase inhibitor mix (ProtAtOnce Ltd, Athens, Greece) at 100x v/v and with Phenylmethanesulfonyl fluoride (PMSF) (MilliporeSigma, MA, USA) at 50x v/v. The samples were maintained at -80^o^C. Before collection, thawed samples were first sonicated and then centrifuged at 2400 rpm for 30 min.

#### Multiplex ELISA – xMAP assays

All lysates were adjusted to a total protein concentration of 250ug/mL. xMap assays were performed on a Luminex FlexMAP 3D platform (Luminex, Austin TX, USA). The customized 17-plex phosphoprotein panel (ProtAtOnce Ltd, Athens, Greece) included: mothers against decapentaplegic homolog-3 (SMAD3), transcription factor AP-1 (JUN), insulin receptor substrate-1 (IRS1), tyrosine-protein phosphatase non-receptor type-11 (PTN11 or SHP2), proline-rich AKT1 substrate-1 (AKTS1), mitogen-activated protein kinase-3 (ERK1 or MK03), RAC-alpha serine/threonine-protein kinase (AKT1), glycogen synthase kinase-3 alpha (GSK3A), heat shock protein beta-1 (HSP27 or HSPβ1), signal transducer and activator of transcription-3 (STAT3), mitogen-activated protein kinase (p38 or MAPK), transcription factor p65 (NFKβ), dual specificity mitogen-activated protein kinase kinase-1 (MEK1 or MP2K1), focal adhesion kinase-1(FAK1), cyclic AMP-responsive element-binding protein-1 (CREB1), nuclear factor erythroid 2-related factor-2 (NRF2 or NF2L2), and 40S ribosomal protein S6 (RS6). For cytokine release measurements a 20-plex antibody assay was developed (ProtAtOnce Ltd, Athens Greece): interleukin 13 (IL13), growth-regulated alpha protein (GROA), interleukin 1a (IL1a), interleukin 8 (IL8), interleukin 12 (IL12), interleukin 6 (IL6), C-C motif chemokine 3 (CCL3), C-X-C motif chemokine 10 (CXCL10), tumour necrosis factor (TNFA), resistin (RETN), ciliary neurotrophic factor (CNTF), C-X-C motif chemokine 11 (CXCL11), transcriptional regulator NRG1 (NRG1), fibroblast growth factor (FGF), metalloproteinase inhibitor 1 (TIMP1), transforming growth factor beta-1 proprotein (TGFB1), C-C motif chemokine 5 (CCL5), C-C motif chemokine 2 (CCL2), matrix metalloproteinase-9 (MMP9) and interleukin 1b (IL1b).

### Quantification and statistical analysis

Statistical analysis of the intracellular lipid accumulation, ROS production and viability assessment was performed using R-programming language. Ordinary one-way ANOVA test with Tukey’s multiple comparisons was performed to compare cell treatments to the respective controls. Biologically relevant comparisons were made between all samples and either the compounds’ diluent (*in vitro* steatosis models, novel steatogenic compounds) or FFAs 200uM + DMSO 0.1% (novel anti-steatogenic compounds). Data are presented as mean ± SEM of at least three independent experiments. Comparisons with a *p-value ≤ 0.05* were considered statistically significant. Calculation of IC_10_ values of the steatogenic compounds was made via 4-parameters logistic regression fitting of the percentage of viability. Heatmaps and volcano plots were created using the R-programming language. Principal component analysis and k-means clustering were also performed using the R-programming language.

## Data Availability

• The original image analysis code generated during this study are available at: https://github.com/BioSysLab/NAFLD_computational_analysis/blob/main/final_program_counting_cores_and_lipids.m https://github.com/BioSysLab/NAFLD_computational_analysis/blob/main/pre_process1.m https://github.com/BioSysLab/NAFLD_computational_analysis/blob/main/createCirclesMask.m
• The code generated for the computational drug repositioning is available at: https://github.com/BioSysLab/NAFLD_computational_analysis/blob/main/gsea_path_analysis_pipeline.R https://github.com/BioSysLab/NAFLD_computational_analysis/blob/main/gene_expression_fig2.R
• The code generated for the statistical analysis of the experimental results is available at: https://github.com/BioSysLab/NAFLD_computational_analysis/blob/main/tukey_final_imaging.R https://github.com/BioSysLab/NAFLD_computational_analysis/blob/main/tukey_final_ROS_DRsteat.R https://github.com/BioSysLab/NAFLD_computational_analysis/blob/main/proteomics.R https://github.com/BioSysLab/NAFLD_computational_analysis/blob/main/PCA.R
