# Supplementary material for: A network-based computational and experimental framework for repurposing compounds toward the treatment of non-alcoholic fatty liver disease
Source: iScience. 2022 Feb 9;25(3):103890. doi: 10.1016/j.isci.2022.103890 (PMC8889147; doi:10.1016/j.isci.2022.103890)
Supplement: Document S1. Figures S1–S20 and Tables S1–S10 [file mmc1.pdf]

iScience, Volume *25*

Supplemental information

A network-based computational and experimental framework for repurposing compounds toward the treatment of non-alcoholic fatty liver disease

Danae Stella Zareifi, Odysseas Chaliotis, Nafsika Chala, Nikos Meimetis, Maria Sofotasiou, Konstantinos Zeakis, Eirini Pantiora, Antonis Vezakis, George K. Matsopoulos, Georgios Fragulidis, and Leonidas G. Alexopoulos

SUPPLEMENTARY INFORMATION

METHODS S1 | IMAGE ANALYSIS | RELATED TO STAR METHODS

The first step of the algorithm is the preprocessing of the images, where a Laplacian filter with a value of -8 in the centre of a 3×3 window with all the other elements defined by value 1 is applied. This filtering is used to sharpen the edges of objects, however, "salt and pepper" noise is added as a consequence of the differential operator. To remove this noise, a Median filter in the 3×3 neighbour area of each pixel is applied. In the processing step, a foreground and a background marker of each image are necessary to separate the objects of interests that are bounded. A Sobel edge mask is used to compute the gradient magnitude of the image, which presents a local maxima at the objects' borders, and minima inside the objects of interest. With the purpose of getting a smoother area in the inner part of the objects, an opening by reconstruction is applied, followed by closing by reconstruction, both with a disc element of 3 pixels radius. By computing the regional maxima of this image a sufficient foreground marker is obtained. The next step is the thresholding of the smoother image that marks the background with Otsu's algorithm. However, the Background marker is too close to the edges of the objects destined for segmentation, and to overcome this obstacle a thinner marker is used by computing the Skeleton by Influence Zones of the foreground, which is the watershed transform of the distance transform of the thresholded image. Finally, the watershed-based segmentation is computed and the objects are labelled, enabling the quantification of the necessary metrics (quantity, area, intensity).

METHODS S2 | IDENTIFYING SIGNIFICANTLY ALTERED PATHWAYS | GENE SET ANALYSIS (GSA) | RELATED TO STAR METHODS

- *"Fisher's combined probability test"*: The basic form of Fisher's tests the independence of multiple variables for $n \times n$ relevance tables by combining p-values from different statistical tests, based on the log-transformed gene-level p-values.
- *"Stouffer's method"*: Stouffer's method combines p-values from different tests using the inverse normal cumulative distribution function and generates Z-scores.
- *"Reporter features"*: The Reporter Features algorithm maps gene analysis to gene networks by identifying groups of adjacent genes that are differentially expressed. This algorithm allows for background distribution correction before calculating the gene set's significance.
- *"Tail strength":* Tail strength (TS) calculates gene set statistics by ranking the genes according to their level of significance and respectively lending the weight, meaning that it is more susceptible to tail deviations.

- *"PAGE"*: This method calculates a Z-score using fold change values for each set of genes and calculates the statistical significance using a normal distribution. (Parametric Analysis of Gene Set Enrichment; page)
- *"maxmean"*: This method calculates the average of the positive and negative expression values for each gene in each set of genes and selects the set of genes with the highest absolute value.
- *"sum", "mean", "median":* These methods calculate the sum, the mean and the median for each set of genes. The former uses the size *t*, while the other two uses the *fold change*.

METHODS S3 | GENE LEVEL STATISTICS OUTPUT GROUPS | RELATED TO STAR METHODS

- *Distinct up*: The genes that are overexpressed and the under-expressed genes are mutated. Overall there is a higher overexpression in the path.
- *Mixed up*: There is a high component of overexpressed genes and the component of under-expressed genes is not taken into account.
- *Non-directional*: The absolute value of the differential expression of the genes is used, as there is a high differential expression of genes, without regard to its direction.
- *Mixed down*: There is a high component of under-expressed genes and the component of overexpressed genes is not taken into account.
- *Distinct down*: The genes that are overexpressed and the under-expressed genes are mutated. Overall there is a higher under-expression in the path.

SUPPLEMENTARY DATA

2.1 NAFLD INDUCTION - IC_{10} CALCULATION

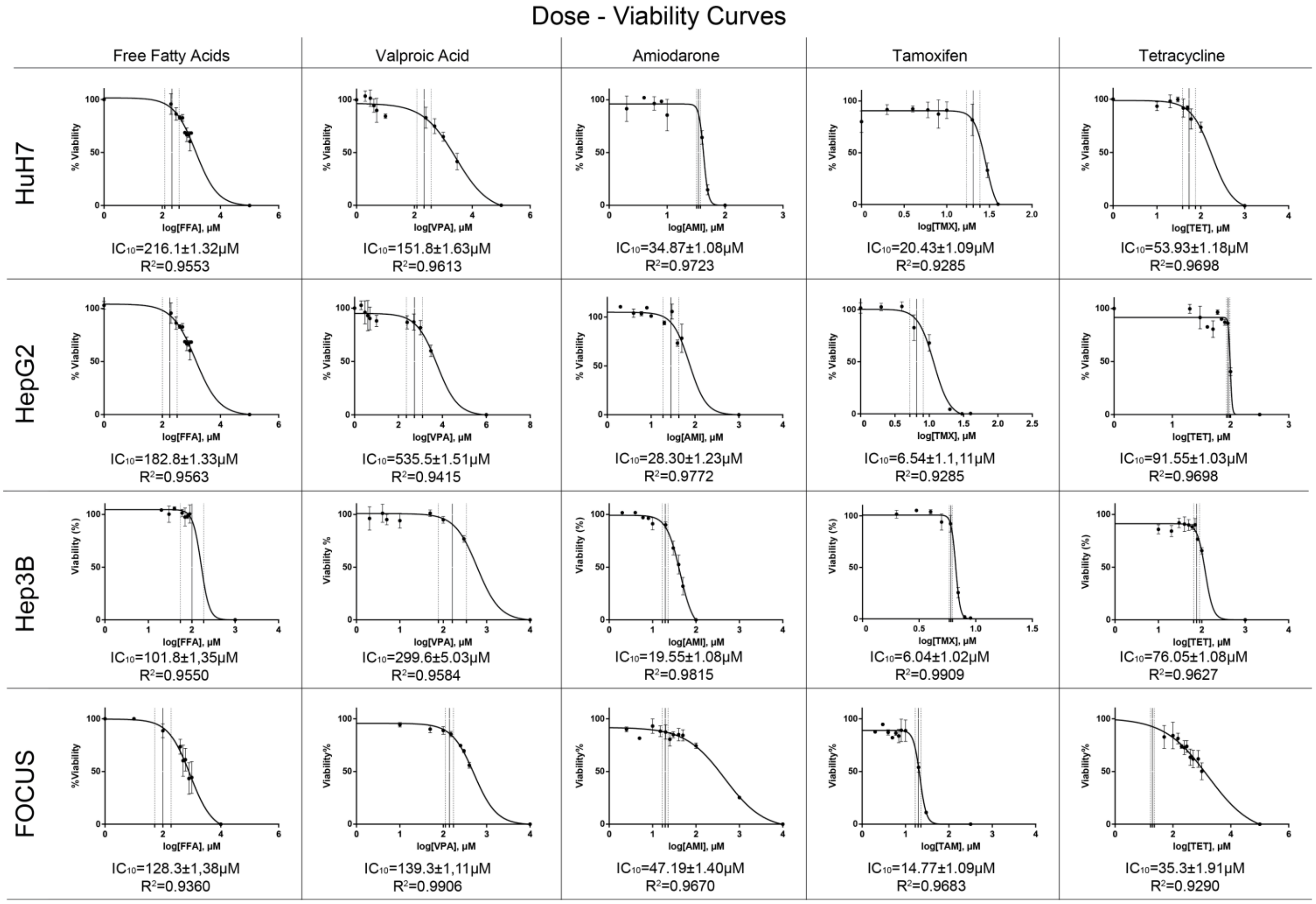

Figure S1. Dose - Cell viability curves and IC_{10} extrapolation. Related to Figure 1 and to Supplemental Figures S2-S4. Cell viability (x-axis) was calculated as the percentage of Relative Fluorescent Units (RFU) of treated cells to untreated cells (control). Data were fitted to a 4 parameters logistic regression model, while the y-axis represents the log10 of concentration, and are presented as mean±SEM of at least three independent experiments. Ticks on the x-axis represent the log10(IC_{10}) ±SEM.

2.2 NAFLD INDUCTION - INTRACELLULAR LIPID ACCUMULATION

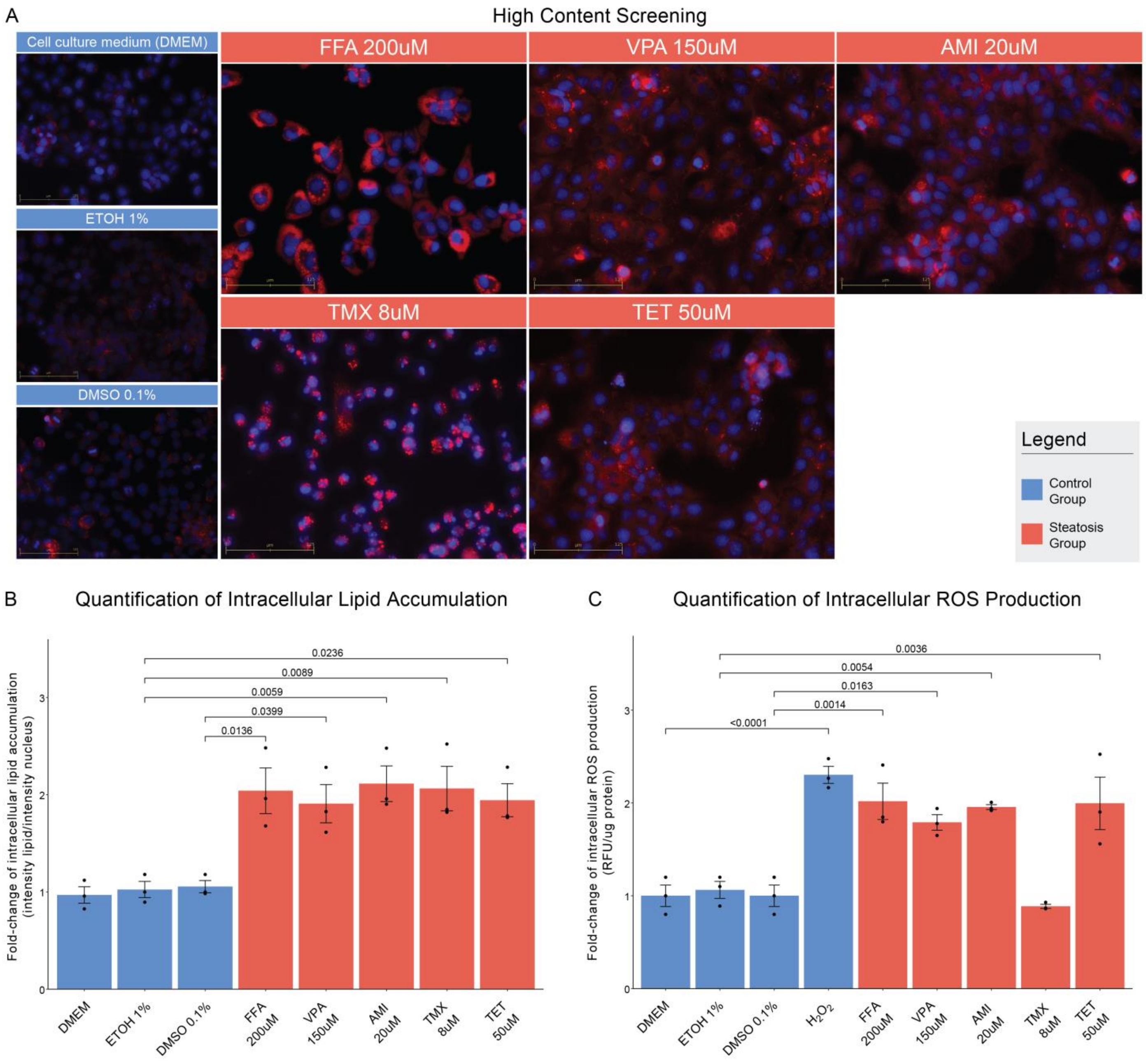

Figure S2. Formation of intracellular lipid droplets and increase of ROS production in HuH7 cells, after treatment with FFA, VPA, AMI, TMX and TET. Related to Figure 1. *A) Intracellular lipid accumulation observed via HCS-based fluorescent microscopy with Nile Red staining. Hoechst33342 was used for staining the nuclei. Images were acquired under 20x optical magnification. B) Quantification of lipid accumulation via MATLAB-based image analysis. Bars represent the FC of lipid droplet intensity per cell in treated cells to the respective controls. C) FC of intracellular ROS production compared to controls. H_2O_2 was used as a positive control. (B, C) Data expressed as mean±SEM of n=3 independent experiments, and (*) denotes p-value ≤ 0.05*

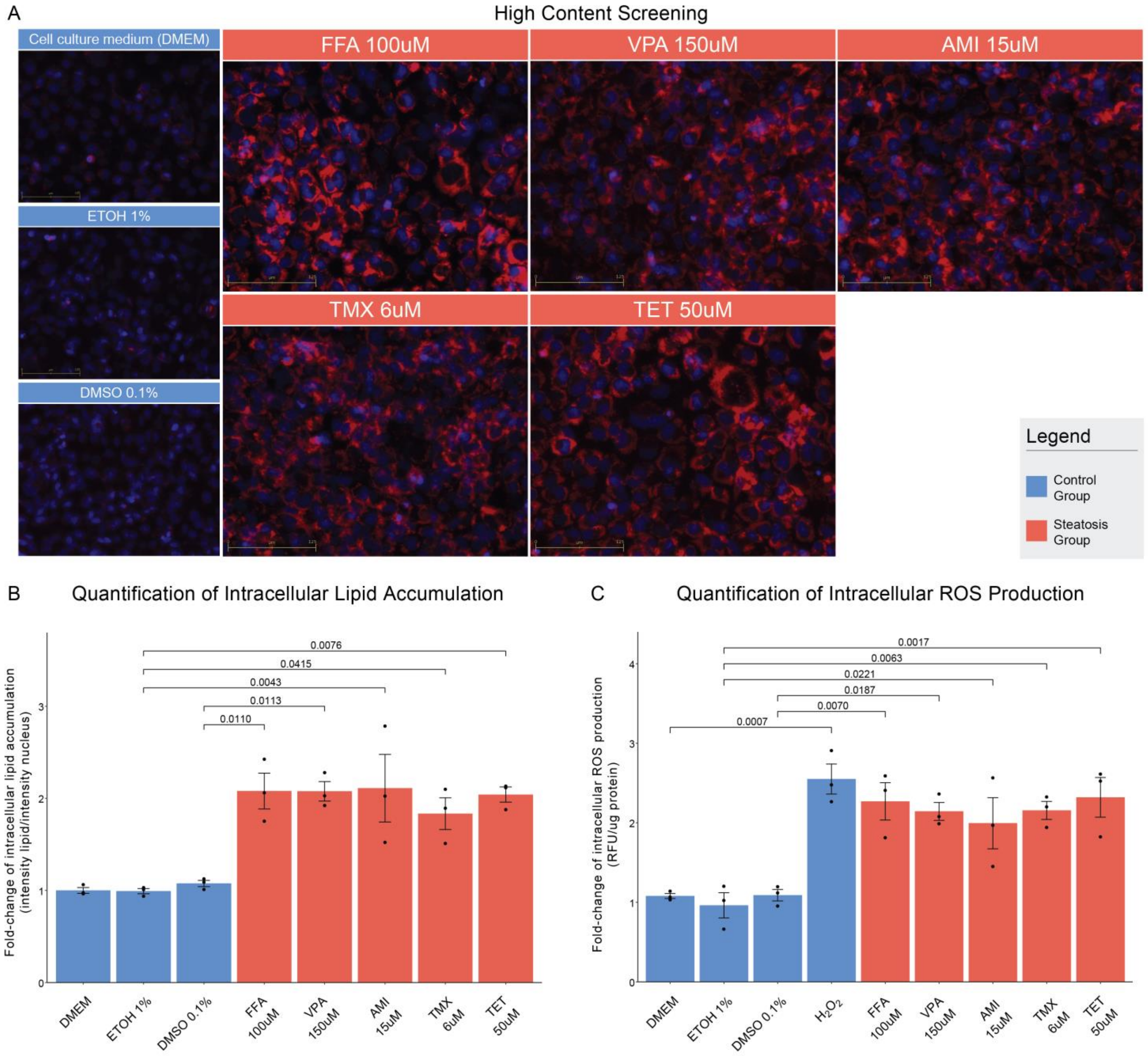

Figure S3. Formation of intracellular lipid droplets and increase of ROS production in Hep3B cells, after treatment with FFA, VPA, AMI, TMX and TET. Related to Figure 1. *A) Intracellular lipid accumulation observed via HCS-based fluorescent microscopy with Nile Red staining. Hoechst33342 was used for staining the nuclei. Images were acquired under 20x optical magnification. B) Quantification of lipid accumulation via MATLAB-based image analysis. Bars represent the FC of lipid droplet intensity per cell in treated cells to the respective controls. C) FC of intracellular ROS production compared to controls. H_2O_2 was used as a positive control. (B, C) Data expressed as mean±SEM of n=3 independent experiments, and (*) denotes p-value ≤ 0.05*

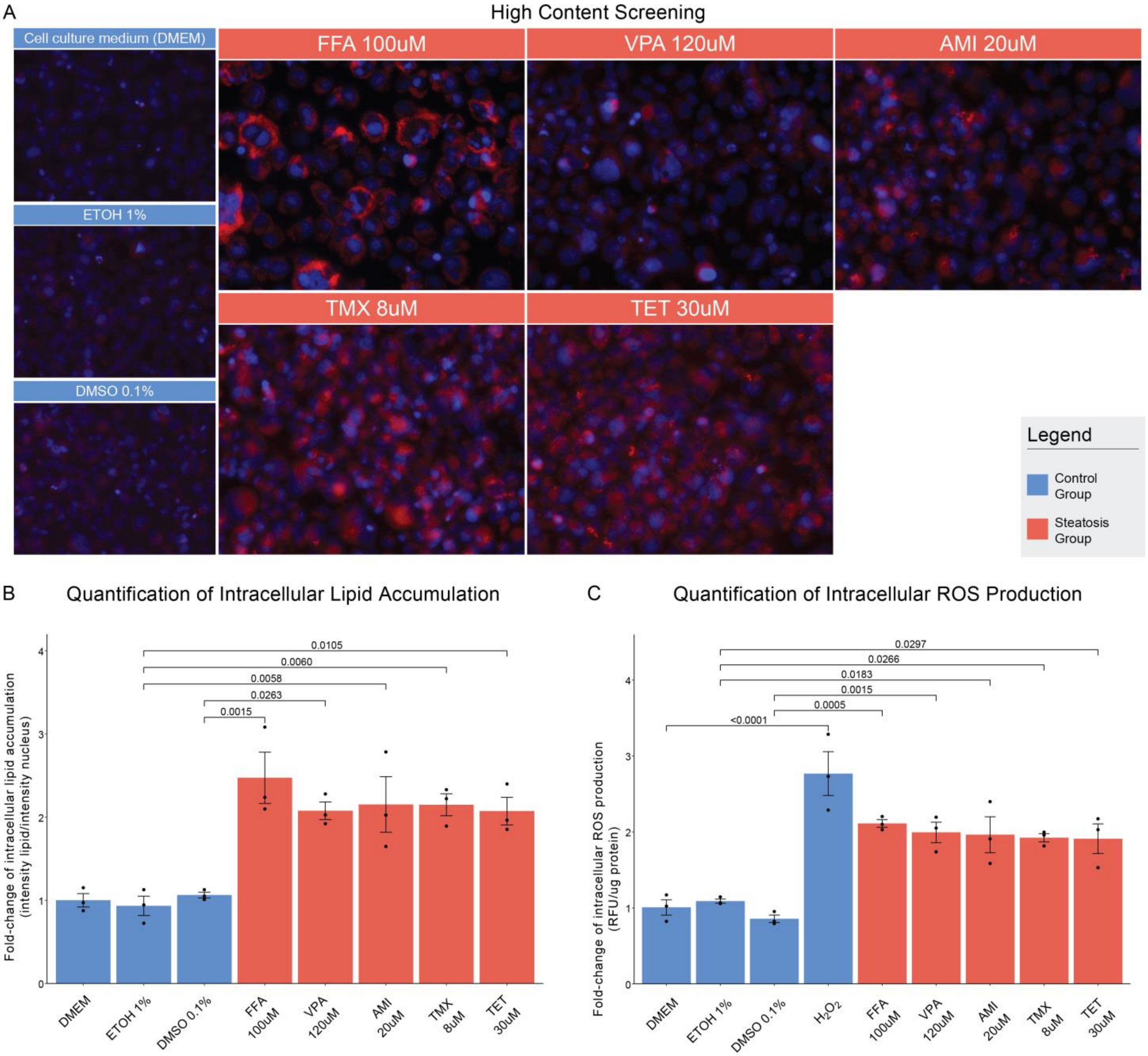

Figure S4. Formation of intracellular lipid droplets and increase of ROS production in FOCUS cells, after treatment with FFA, VPA, AMI, TMX and TET. Related to Figure 1. *A) Intracellular lipid accumulation observed via HCS-based fluorescent microscopy with Nile Red staining. Hoechst33342 was used for staining the nuclei. Images were acquired under 20x optical magnification. B) Quantification of lipid accumulation via MATLAB-based image analysis. Bars represent the FC of lipid droplet intensity per cell in treated cells to the respective controls. C) FC of intracellular ROS production compared to controls. H_2O_2 was used as a positive control. (B, C) Data expressed as mean±SEM of n=3 independent experiments, and (*) denotes p-value ≤ 0.05*

2.5 PATHWAYS AFFECTED

Drug/Compound	Gene	Pathway
**Valproic acid**	ACADSB	Fatty acid metabolism
		Metabolism of amino acids and derivatives
	OGDH	Metabolism of amino acids and derivatives
	PPARA	PPAR signalling
		Nuclear receptors
		Metabolism of lipids and lipoproteins
		Fatty acid, triacylglycerol and ketone body metabolism
	PPARD	PPAR signalling
		Nuclear receptors
	PPARG	PPAR signalling
		Nuclear receptors
		NFAT transcription factor
		Metabolism of lipids and lipoproteins
		Fatty acid, triacylglycerol and ketone body metabolism
**Amiodarone**	ADRB1	Endocytosis
	PPARG	PPAR signalling
		Nuclear receptors
		NFAT transcription factor
		Metabolism of lipids and lipoproteins
		Fatty acid, triacylglycerol and ketone body metabolism
**Tetracycline**	*X*	**Pathways affected by the drug did not overlap with pathways deregulated in the clinical data*
**Tamoxifen**	ESR1	Activating Transcription Factor 2
		Activator Protein 1
	EBP	Metabolism of lipids and lipoproteins
**Oleic acid**	PPARA	PPAR signalling
		Nuclear receptors
		Metabolism of lipids and lipoproteins
		Fatty acid, triacylglycerol and ketone body metabolism
	PPARD	PPAR signalling
		Nuclear receptors
	PPARG	PPAR signalling
		Nuclear receptors
		NFAT transcription factor
		Metabolism of lipids and lipoproteins
		Fatty acid, triacylglycerol and ketone body metabolism
	RXRA	PPAR signalling
		Metabolism of lipids and lipoproteins
		Fatty acid, triacylglycerol and ketone body metabolism
**Palmitic acid**	PPT1	Lysosome
	PPARA	PPAR signalling
		Nuclear receptors
		Metabolism of lipids and lipoproteins
		Fatty acid, triacylglycerol and ketone body metabolism

Table S1. Pathways affected that belong to the intersection of the “Clinical Data Pathway Group” and “Steatogenic Compounds Pathway Group”. Related to Figure 3.

2.6 DIFFERENTIAL GENE EXPRESSION ON TARGET-PATHWAYS

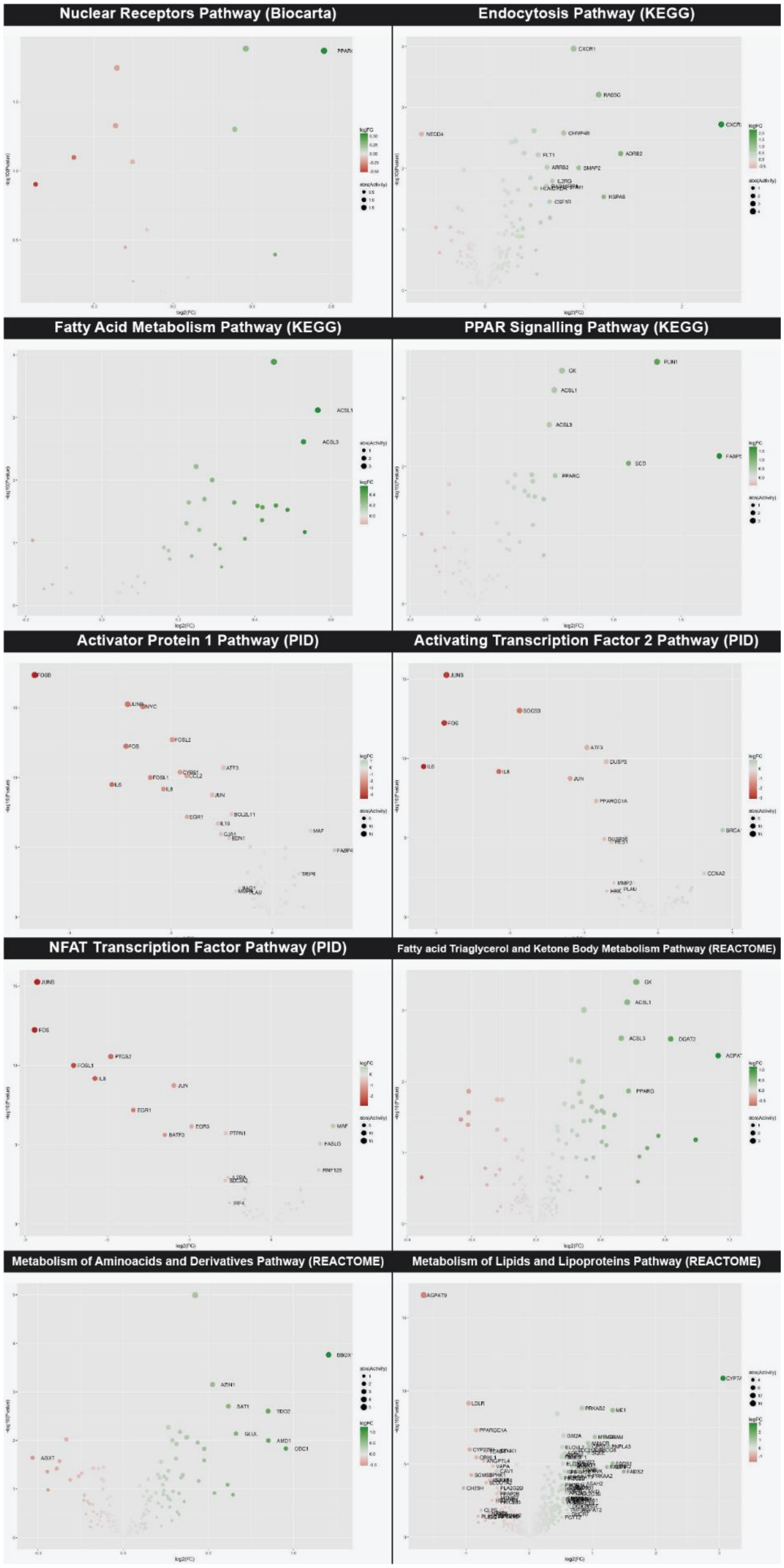

Figure S5. Differential gene expression based on clinical data obtained from GEO-NCBI for the selected pathways to target for the treatment of NAFLD/NASH. Related to Figure 3. *Differential gene expression data are presented in volcano plots, denoting the most statistically significant differentially expressed genes. Each point on the plot corresponds to a gene, while the y-axis represents the negative decimal log of* $p - value$ *and the x-axis represents* $log_2 FC$. *The higher the gene on the vertical axis, the more statistically significant the differential expression, and the farther from zero on the horizontal axis, the greater the intensity.*

2.7 CMAP RESULTS

Tables presenting the drugs inferred by cMap for every "signature question'. For each steatogenic compound used as "signature question" two tables are presented, one for compounds that have opposite gene signatures and one for compounds with similar gene signatures. N represents the number of independent experiments included in cMap for each compound. Enrichment score takes values [-1,1] and is a meter of similarity of the gene expression of cell lines included in cMap after treatment with a compound. Positive values correspond to similar gene expression, whilst negative values correspond to opposite gene expression. Specificity value is a meter for evaluating the singularity of the given compound with the "signature question", the higher the value the most common the connection between the two compounds. The %Non-null value signifies the percentage of experiments for a given compound in accordance with the enrichment score.

1. Amiodarone

Cmap name	Mean	n	Enrichment Score (ES)	P	Specificity	% non null
**Timolol**	-0.510	4	-0.792	0.00376	0.0224	100
**Ketoconazole**	-0.408	4	-0.689	0.0213	0.0000	75
**Aminoglutethimide**	-0.280	3	-0.781	0.02157	0.0179	66
**Fusidic acid**	-0.406	4	-0.666	0.02801	0.0063	75

Table S2. Drugs with reverse gene signature compared to Amiodarone. Related to Figure 3.

Cmap name	Mean	n	Enrichment Score (ES)	P	Specificity	% non null
**Ethisterone**	0.432	6	0.698	0.00205	0.0000	8
**Meclizine**	0.412	5	0.709	0.00491	0.0131	80
**Omeprazole**	0.379	4	0.739	0.00889	0.0642	100
**Trimetazidine**	0.338	4	0.720	0.01243	0.0078	100
**Prednisone**	0.282	5	0.607	0.02842	0.0526	80
**Amiloride**	0.340	5	0.597	0.03276	0.0167	80
**Ursodeoxycholic acid**	0.390	3	0.725	0.04054	0.0517	100

Table S3. Drugs with similar gene signature compared to Amiodarone. Related to Figure 3. Tamoxifen

Cmap name	Mean	n	Enrichment Score (ES)	P	Specificity	% non null
**Estropipate**	-0.374	4	-0.816	0.00209	0.0000	50
**Physostigmine**	-0.391	4	-0.804	0.00284	0.0000	75
**Cefmetazole**	-0.312	4	-0.708	0.01514	0.0142	50
**Carbimazole**	-0.396	3	-0.792	0.01827	0.0480	66
**Sulindac**	-0.342	7	-0.524	0.02499	0.0388	57
**Diflorasone**	-0.376	4	-0.668	0.02731	0.0186	50

Table S4. Drugs with reverse gene signature compared to Tamoxifen. Related to Figure 3.

Cmap name	Mean	n	Enrichment Score (ES)	P	Specificity	% non null
**Mefloquine**	0.476	5	0.933	0.00000	0.0098	100
**Sirolimus**	0.215	4 4	0.330	0.00012	0.2590	65
**Acepromazine**	0.360	4	0.810	0.00241	0.0074	100
**Disulfiram**	0.357	5	0.709	0.00495	0.0694	80
**Clomifene**	0.415	4	0.748	0.00778	0.0517	100
**Ivermectin**	0.321	5	0.651	0.01442	0.1075	80
**Clomipramine**	0.410	4	0.705	0.01578	0.1105	100
**Albendazole**	0.390	3	0.793	0.01815	0.0116	100
**Rimexolone**	0.333	4	0.689	0.01950	0.0840	100
**Gallamine triethiodide**	0.314	5	0.616	0.02453	0.0081	80
**Clotrimazole**	0.263	5	0.597	0.03258	0.1333	80
**Pimozide**	0.325	4	0.642	0.03995	0.1708	75
**Fenoterol**	0.326	3	0.723	0.04202	0.0692	100
**Isoetarine**	0.230	4	0.637	0.04227	0.0400	75
**Progesterone**	0.282	4	0.632	0.04579	0.0307	75
**Mifepristone**	0.241	4	0.629	0.04768	0.0584	75

Table S5. Drugs with similar gene signature compared to Tamoxifen. Related to Figure 3.

1. Tetracycline

Cmap name	Mean	n	Enrichment Score (ES)	P	Specificity	% non null
**Raloxifen**	-0.380	7	-0.519	0.02697	0.0520	57
**Oxprenolol**	-0.502	4	-0.650	0.03529	0.0594	75
**Dipivefrin**	-0.519	4	-0.641	0.03965	0.0526	75
**Metoprolol**	-0.417	4	-0.624	0.04953	0.0534	75

Table S6. Drugs with reverse gene signature compared to Tetracycline. Related to Figure 3.

Cmap name	Mean	n	Enrichment Score (ES)	P	Specificity	% non null
**Aceclofenac**	0.720	4	0.727	0.01108	0.0137	75
**Cefotaxime**	0.675	5	0.592	0.03475	0.0136	80

**Cortisone**	0.701	3	0.738	0.03523	0.0826	100

Table S7. Drugs with similar gene signature compared to tetracycline. Related to Figure 3.

2. Valproic acid

Cmap name	Mean	n	Enrichment Score (ES)	P	Specific ity	% non null
**Naftifine**	-0.558	4	-0.698	0.01751	0.0196	75
**Ketoprofen**	-0.370	6	-0.525	0.04660	0.0385	50
**Pralidoxime**	-0.320	4	-0.625	0.04864	0.0526	75

Table S8. Drugs with reverse gene signature compared to Valproic acid. Related to Figure 3.

Cmap name	Mean	n	Enrichment	P	Specific ity	% non null
**Resveratrol**	0.294	9	0.655	0.00026	0.1226	77
**Nabumetone**	0.267	4	0.722	0.01182	0.0130	100
**Mepacrine**	0.330	2	0.860	0.03941	0.0677	100

Table S9. Drugs with similar gene signature compared to Valproic acid. Related to Figure 3.

2.8 REPOSITIONING COMPOUNDS PATHWAYS

#	Drug/Compound	Gene	Pathway
1	**Naftifine**	SQLE	Metabolism of lipids and lipoproteins
2	**Ketoprofen**	PTGS2	NFAT transcription factor
		CXCR1	Endocytosis
3	**Pralidoxime**	ACHE	Activating Transcription Factor 2
			Metabolism of lipids and lipoproteins
4	**Resveratrol**	PTGS2	NFAT transcription factor
		PI4K2B	Metabolism of lipids and lipoproteins
		NR1L3	Nuclear receptors
		PPARA	PPAR signalling
			Nuclear receptors
			Metabolism of lipids and lipoproteins
			Fatty acid, triacylglycerol and ketone body metabolism
		PPARG	PPAR signalling
			Nuclear receptors
			NFAT transcription factor
			Metabolism of lipids and lipoproteins
			Fatty acid, triacylglycerol and ketone body metabolism
5	**Nabumetone**	PTGS2	NFAT transcription factor
6	**Quinacrine**	PLA2G6	Metabolism of lipids and lipoproteins
		PLA2G4	
7	**Timolol**	ADRB1	Endocytosis
8	**Ketoconazole**	CYP21A2	Metabolism of lipids and lipoproteins
		NR1L3	Nuclear receptors
9	**Aminoglutethimide**	CYP19A1	Metabolism of lipids and lipoproteins
		CYP11A1	
10	**Fusidic acid**	ABCB11	Nuclear receptors
			Metabolism of lipids and lipoproteins
11	**Meclizine**	NR1L3	Nuclear receptors

12	**Estradiol**	ESR1	Activating Transcription Factor 2
			Activator Protein 1
		WCOA2	Metabolism of lipids and lipoproteins
			Fatty acid, triacylglycerol and ketone body metabolism
13	**Omeprazole**	ABCB3	Nuclear receptors
			Metabolism of lipids and lipoproteins
14	**Trimetazidine**	ACAA1	PPAR signalling
			Metabolism of lipids and lipoproteins
			Fatty acid, triacylglycerol and ketone body metabolism
15	**Prednisone**	NR3C1	Activator Protein 1
		HSD11B1	Metabolism of lipids and lipoproteins
		ABCB1	Nuclear receptors
		SLCOA2	Metabolism of lipids and lipoproteins
16	**Amiloride**	PLAU	Activator Protein 1
			Activating Transcription Factor 2
17	**Ursodeoxycholic acid**	ABCB1	Nuclear receptors
			Metabolism of lipids and lipoproteins
		SLCO1A1	Metabolism of lipids and lipoproteins
		SLCO1A2	
		NR1H4	Nuclear receptors
18	**Raloxifene**	ESR1	Activating Transcription Factor 2
			Activator Protein 1
19	**Oxprenolol**	ADRB1	Endocytosis
		ADRB2	
		ADRB3	
20	**Mifepristone**	NR3C1	Activator Protein 1
		ABCB1	Nuclear receptors
21	**Fenoterol**	ADRB1	Endocytosis
		ADRB2	
22	**Dipivrefin**	ADRB2	Endocytosis
		ACHE	Activating Transcription Factor 2
			Metabolism of lipids and lipoproteins
23	**Metoprolol**	ADRB1	Endocytosis
		ADRB2	Endocytosis
		ABCB1	Nuclear receptors
			Metabolism of lipids and lipoproteins
24	**Aceclofenac**	PTGS2	NFAT transcription factor
25	**Cefotaxime**	ALB	Metabolism of lipids and lipoproteins
26	**Hydrocortisone**	HSD3B1	Metabolism of lipids and lipoproteins
		SLCO1A2	
27	**Cortisone acetate**	NR3C1	Activator Protein 1
28	**Estrone sulfate**	ESR1	Activating Transcription Factor 2
			Activator Protein 1
29	**Physostigmine**	ACHE	Activating Transcription Factor 2
			Metabolism of lipids and lipoproteins
30	**Cefmetazole**	ALB	Metabolism of lipids and lipoproteins
	**Carbimazole**	TPO	Metabolism of amino acids and derivatives
31	**Sulindac**	AKR1B1	Metabolism of lipids and lipoproteins
		PPARD	PPAR signalling
			Nuclear receptors
32	**Diflorasone**	NR3C1	Activator Protein 1

33	**Mefloquine**	ACHE	Activating Transcription Factor 2
			Metabolism of lipids and lipoproteins
		ABCB1	Nuclear receptors
			Metabolism of lipids and lipoproteins
34	**Sirolimus**	SLCO1B1	Metabolism of lipids and lipoproteins
		ABCB1	Nuclear receptors
			Metabolism of lipids and lipoproteins
35	**Acepromazine**	ALB	Metabolism of lipids and lipoproteins
36	**Disulfiram**	DBH	Metabolism of lipids and lipoproteins
37	**Clomifene**	ESR1	Activating Transcription Factor 2
			Activator Protein 1
		ABCB1	Nuclear receptors
			Metabolism of lipids and lipoproteins
38	**Ivermectin**	ABCB1	Nuclear receptors
			Metabolism of lipids and lipoproteins
39	**Clomipramin**	ALB	Metabolism of lipids and lipoproteins
		ABCB1	Nuclear receptors
			Metabolism of lipids and lipoproteins
40	**Albendazole**	ABCB1	Nuclear receptors
			Metabolism of lipids and lipoproteins
41	**Rimexolone**	NR3C1	Activator Protein 1
42	**Gallamine triethiotide**	ACHE	Activating Transcription Factor 2
			Metabolism of lipids and lipoproteins
43	**Pimozide**	ABCB1	Nuclear receptors
			Metabolism of lipids and lipoproteins
44	**Clotrimazole**	NR1L3	Nuclear receptors
		ABCB1	Nuclear receptors
			Metabolism of lipids and lipoproteins
		ABCB3	Nuclear receptors
			Metabolism of lipids and lipoproteins
45	**Progesterone**	ESR1	Activating Transcription Factor 2
			Activator Protein 1
		ABCB1	Nuclear receptors
			Metabolism of lipids and lipoproteins
		SLC10A1	Metabolism of lipids and lipoproteins
46	**Isoetharine**	ADRB1	Endocytosis
		ADRB2	

Table S10. The target-genes and affected pathways significantly differentiated in NAFLD/NASH, as proposed by the DR platform, of the repositioned compounds. Related to Figure 3.

2.9 HCS OF REPOSITIONED COMPOUNDS

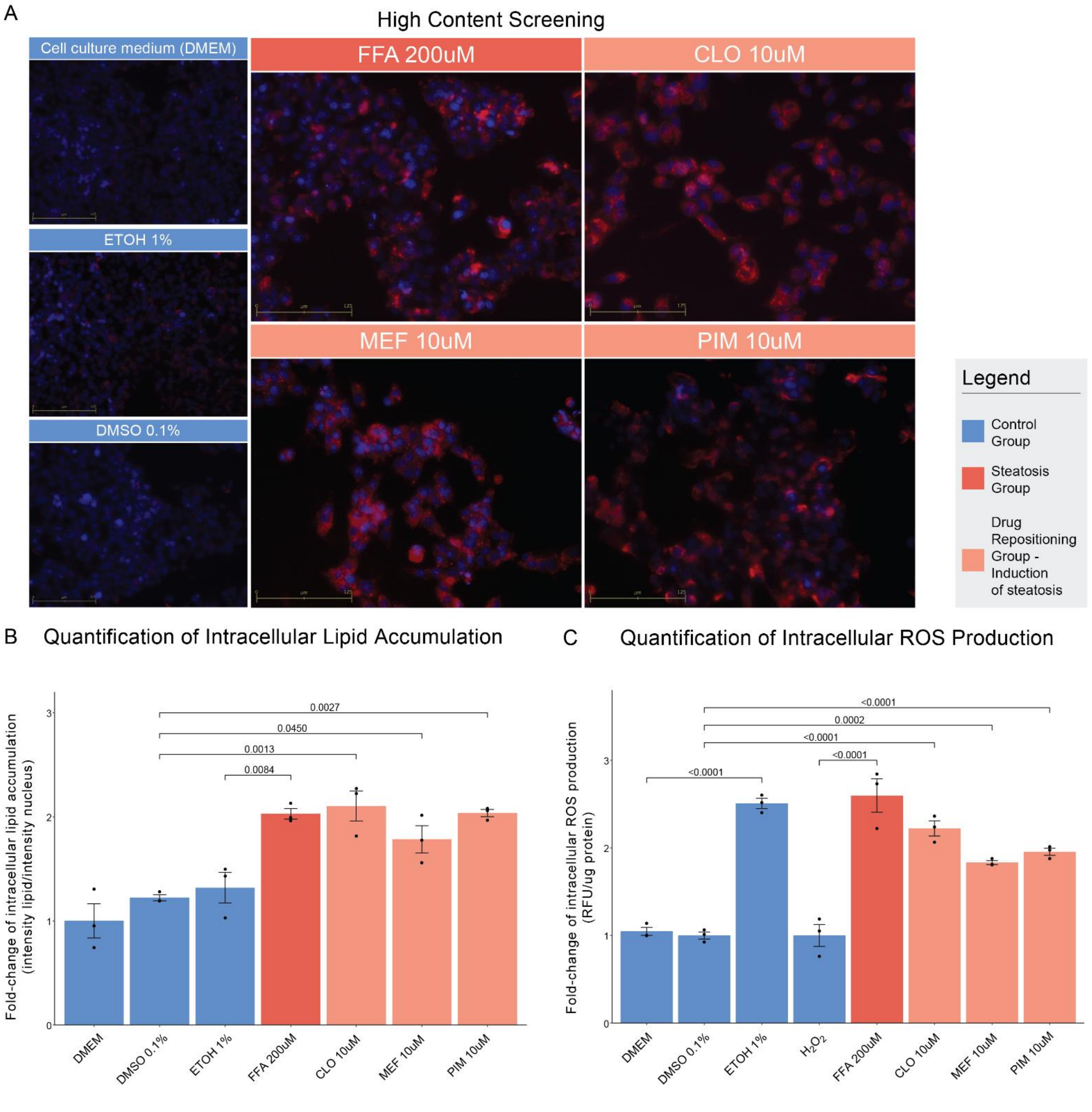

Figure S6. Formation of intracellular lipid droplets and increase of ROS production in HepG2 cells, after treatment with FFA, CLO, MEF and PIM. Related to Figure 4. *A) Intracellular lipid accumulation observed via HCS-based fluorescent microscopy with Nile Red staining. Hoechst33342 was used for staining the nuclei. Images were acquired under 20x optical magnification. B) Quantification of lipid accumulation via MATLAB-based image analysis. Bars represent the FC of lipid droplet intensity per cell in treated cells over respective controls. C) FC of intracellular ROS production compared to controls. H_2O_2 was used as a positive control. (B, C) Data expressed as mean±SEM of n=3 independent experiments, and the p-value is denoted by brackets.*

2.10 HCS OF REPOSITIONED COMPOUNDS

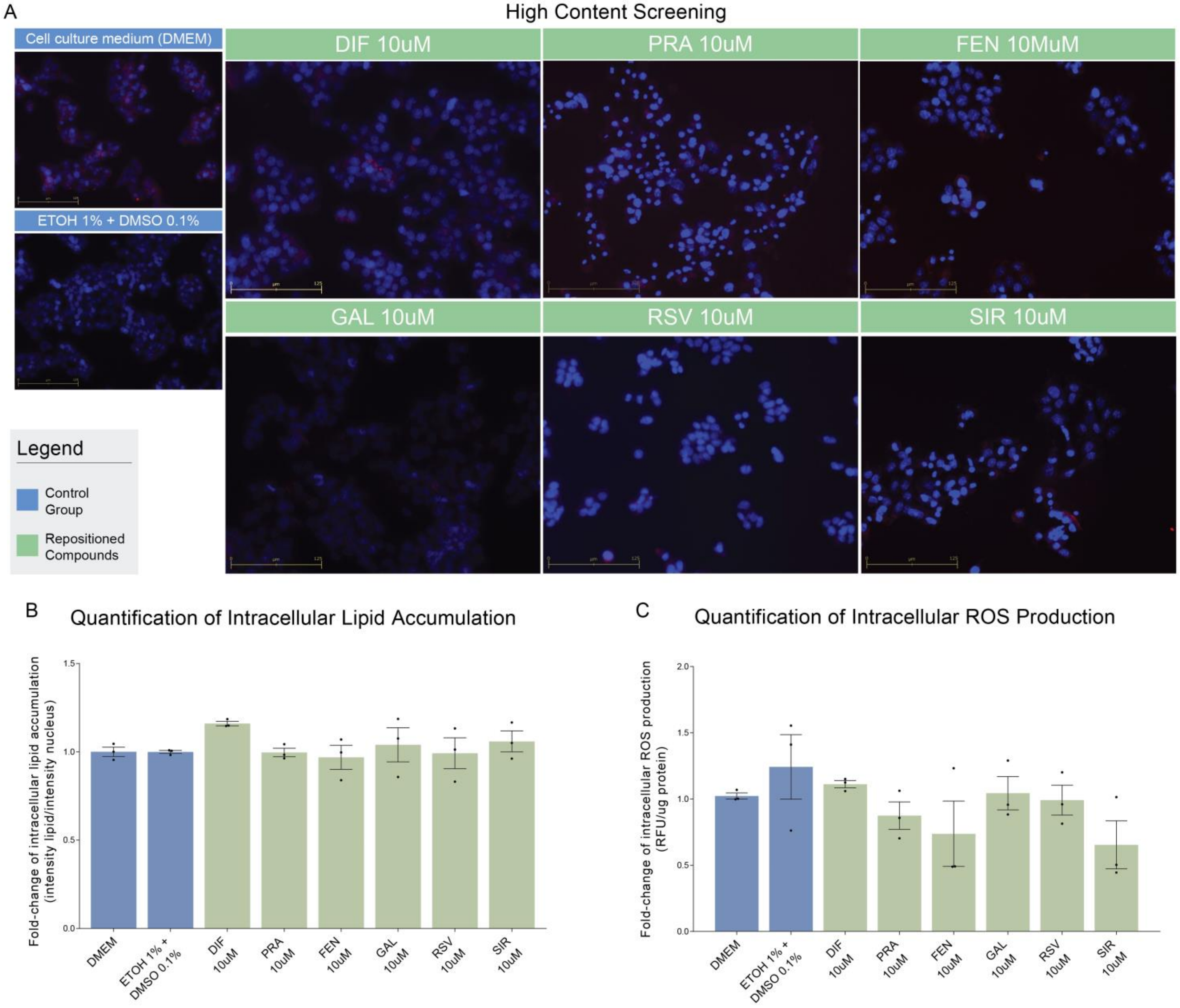

Figure S7. Treatment with the repositioned compounds alone does not increase intracellular lipid accumulation and oxidative stress. Related to Figure 4. *A) Intracellular lipid accumulation observed via HCS-based fluorescent microscopy with Nile Red staining. Hoechst33342 was used for staining the nuclei. Images were acquired under 20x optical magnification. B) Quantification of lipid accumulation via MATLAB-based image analysis. Bars represent the FC of lipid droplet intensity per cell in treated cells to the respective controls. C) FC of intracellular ROS production compared to controls.* H_2O_2 *was used as a positive control. (B, C) Data expressed as mean±SEM of n=3 independent experiments.*

2.11 NETWORK REPRESENTATION OF THE GENES AND PATHWAYS-TO-TARGET AFFECTED BY THE REPUPOSED COMPOUNDS

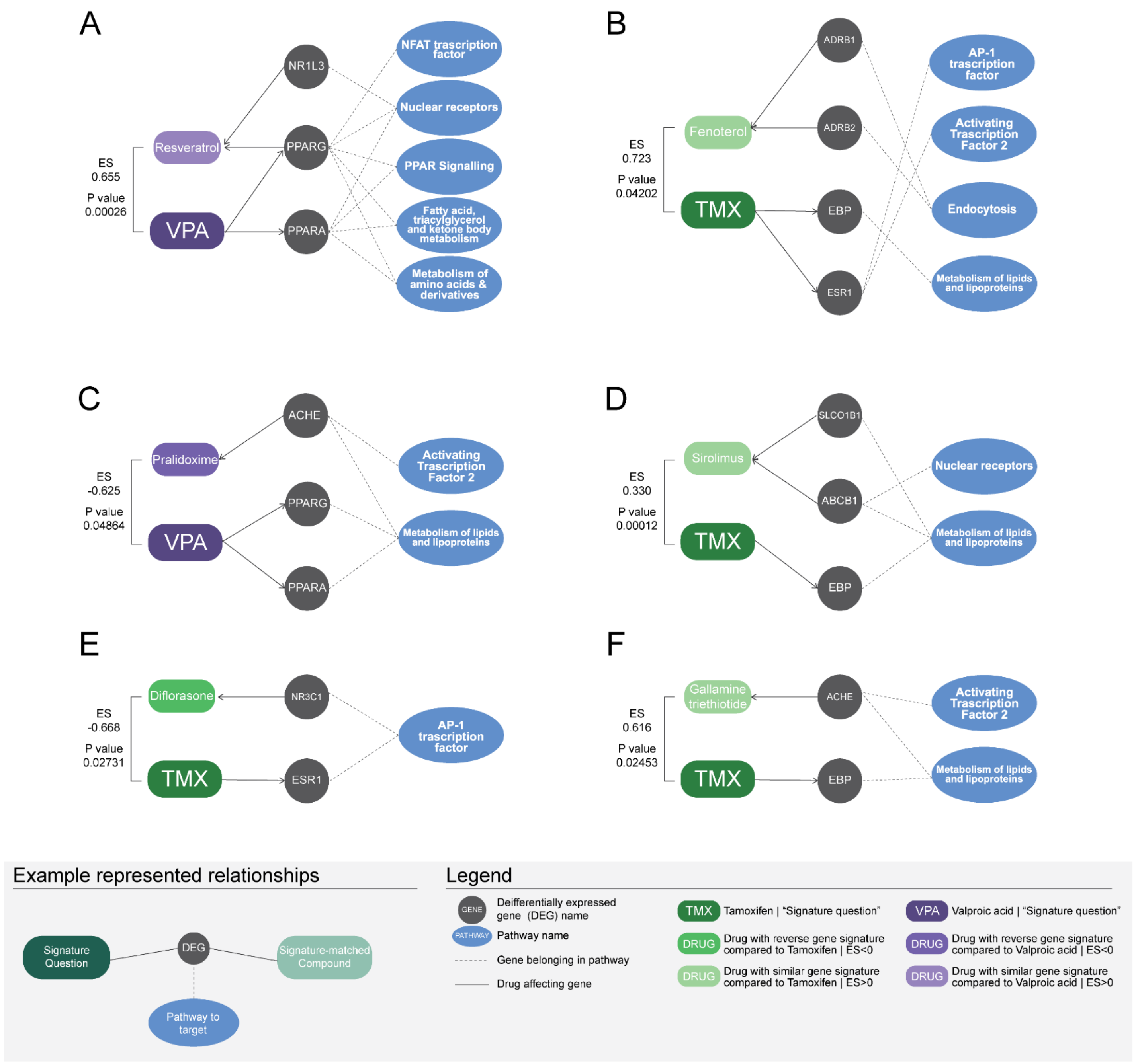

Figure S8. Network representation of the identification process of the resulting repurposed compounds, as proposed by the DR platform. Related to Figure 3. *The target-pathways depicted in blue in the centre of the graph belong at the intersection of the "Clinical Data Pathway Group" and "Steatogenic Compounds Pathway Group". The differentially expressed genes (DEGs) and the pathways affected by each compound are illustrated in grey circles. Each steatosis-inducing compound was used as a "signature question" to cMap. The cMap tool compares two-sample distributions using the Kolmogorov-Smirnov (K-S) statistical test and calculates an Enrichment Score that takes values in the interval [-1,1]. ES>0 signifies that two drugs present similar gene signature, while ES<0 means that two drugs have reverse gene*

signatures. The steatosis-inducing compounds are depicted in rectangles of different colours. Every steatosis-inducing compound drugs with ES>0 or ES<0 is illustrated with rectangles of the same colour scale. Lines connect each compound and drug with their target-genes and the pathways they affect.

2.12 STATISTICAL ANALYSIS OF THE PROTEOMIC MEASUREMENTS

Bar graphs of phospho-proteomic and cytokine release measurements per repositioned compound and the results of the statistical analysis conducted are summarized in Supplementary Figures S9-S20. Fold change of the Median Fluorescent Intensity (MFI) of each treatment to the respective controls was calculated and results were analyzed using regular two-way ANOVA with Tukey's multiple comparisons test.

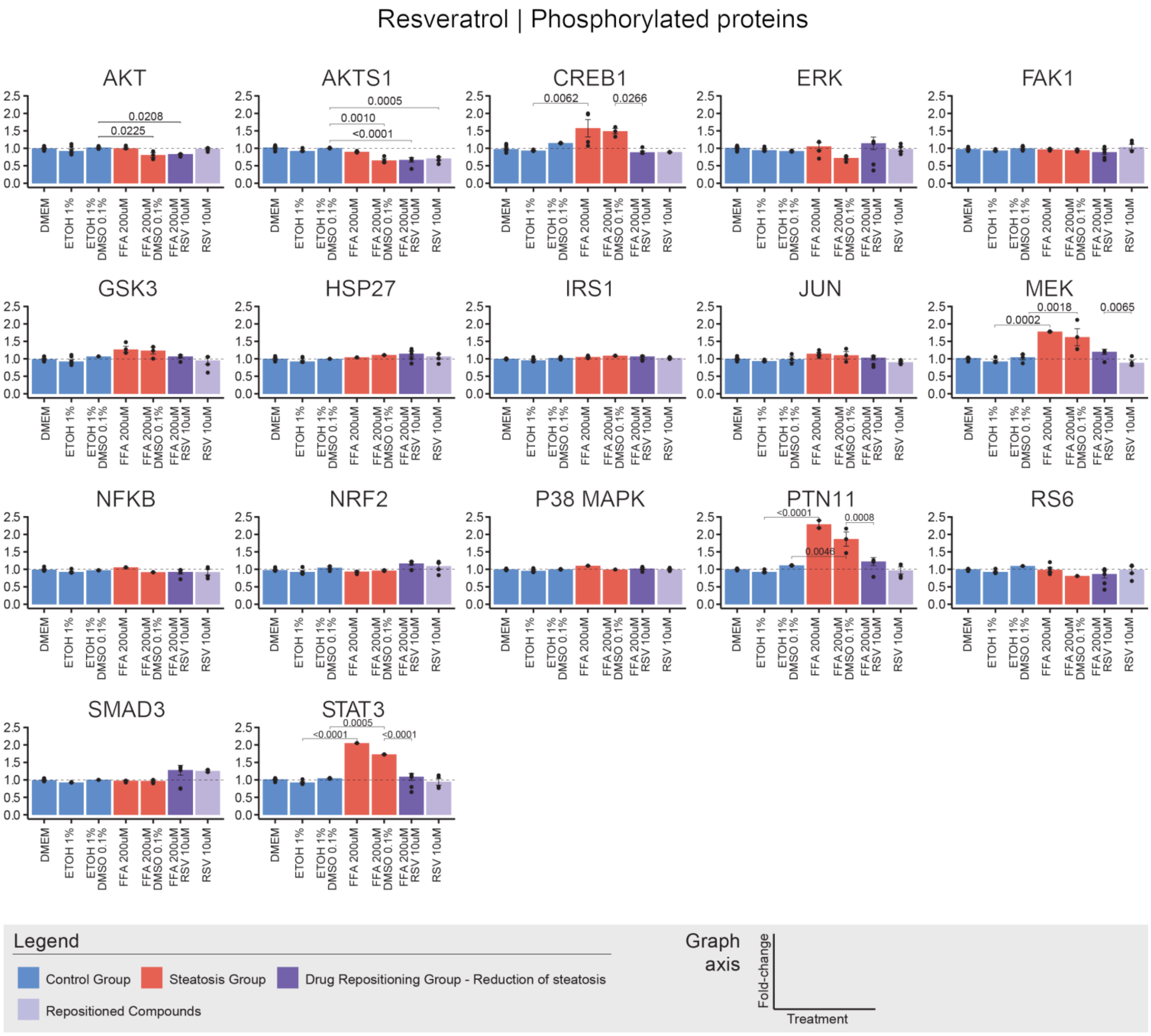

Figure S9 Statistical analysis of the effect of Resveratrol on the phosphorylated proteins measured. Related to Figure 5. *Bars represent the FC of median fluorescent intensity per protein in treated cells over respective controls. Data expressed as mean±SEM of at least n=3 independent experiments, and the p-value is denoted by brackets.*

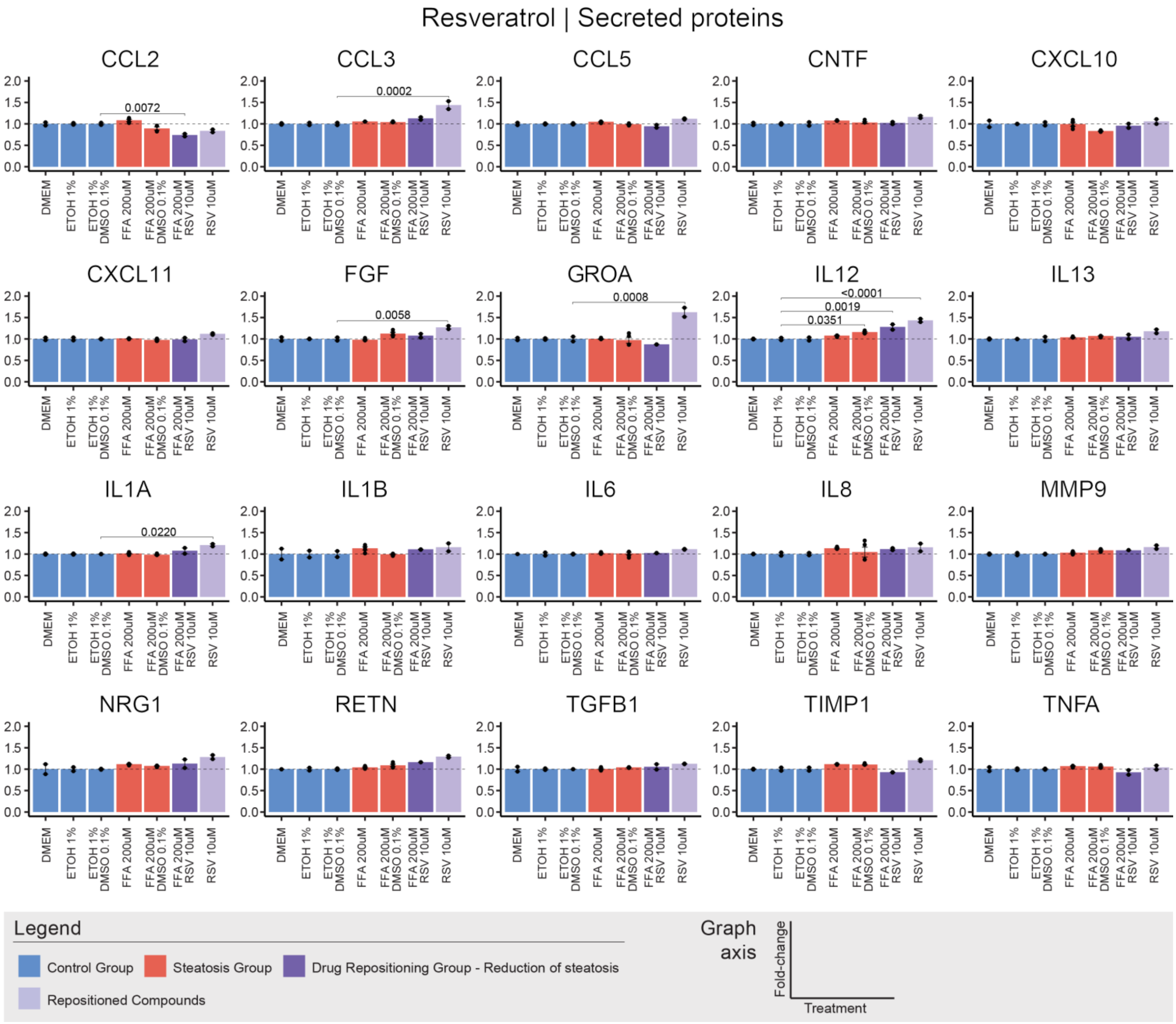

Figure S10 Statistical analysis of the effect of Resveratrol on the secreted proteins measured. Related to Figure 5. *Bars represent the FC of median fluorescent intensity per protein in treated cells over respective controls. Data expressed as mean±SEM of at least n=3 independent experiments, and the p-value is denoted by brackets.*

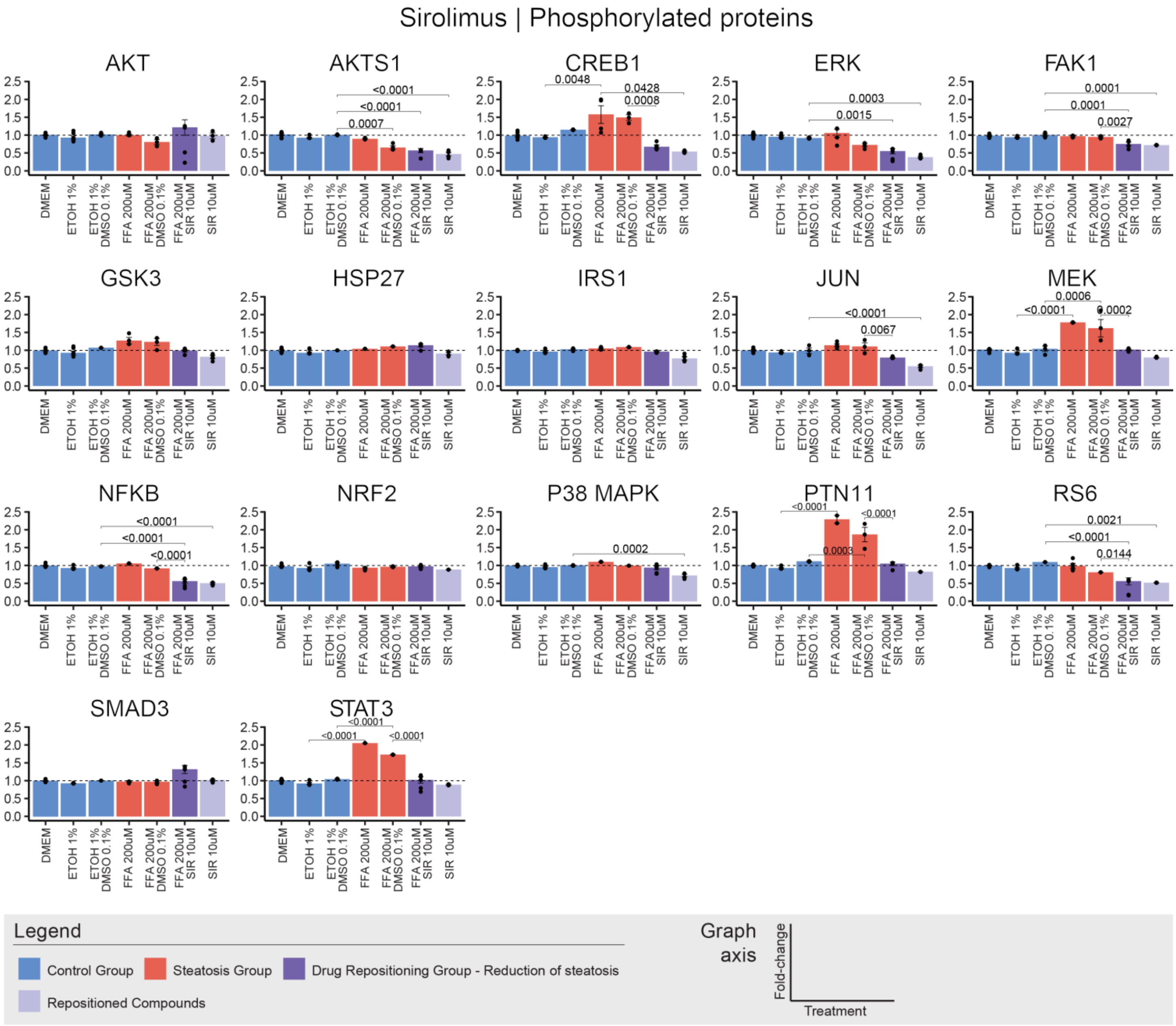

Figure S11 Statistical analysis of the effect of Sirolimus (rapamycin) on the phosphorylated proteins measured. Related to Figure 5. *Bars represent the FC of median fluorescent intensity per protein in treated cells over respective controls. Data expressed as mean±SEM of at least n=3 independent experiments, and the p-value is denoted by brackets.*

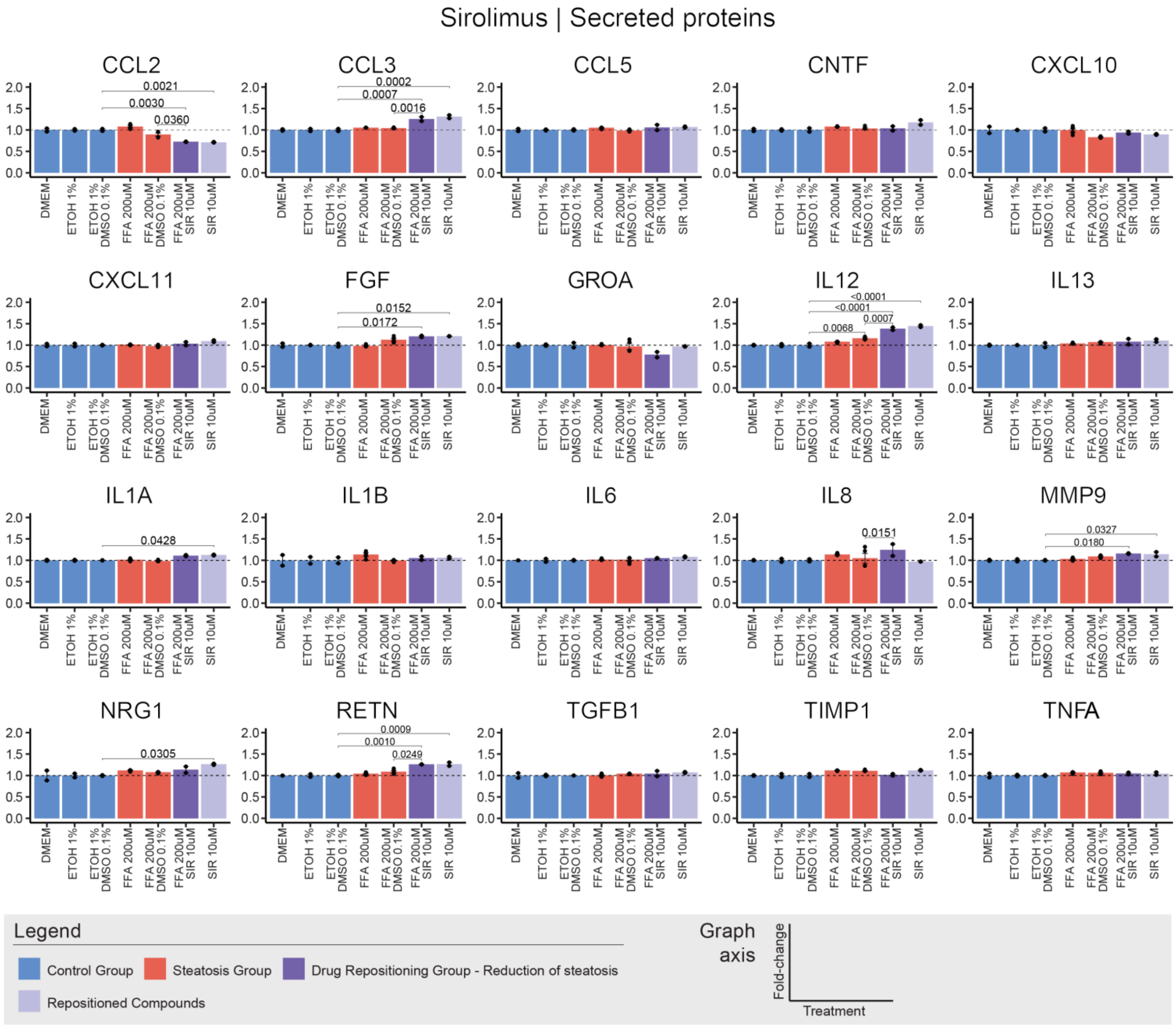

Figure S12 Statistical analysis of the effect of Sirolimus (rapamycin) on the secreted proteins measured. Related to Figure 5. *Bars represent the FC of median fluorescent intensity per protein in treated cells over respective controls. Data expressed as mean±SEM of at least n=3 independent experiments, and the p-value is denoted by brackets.*

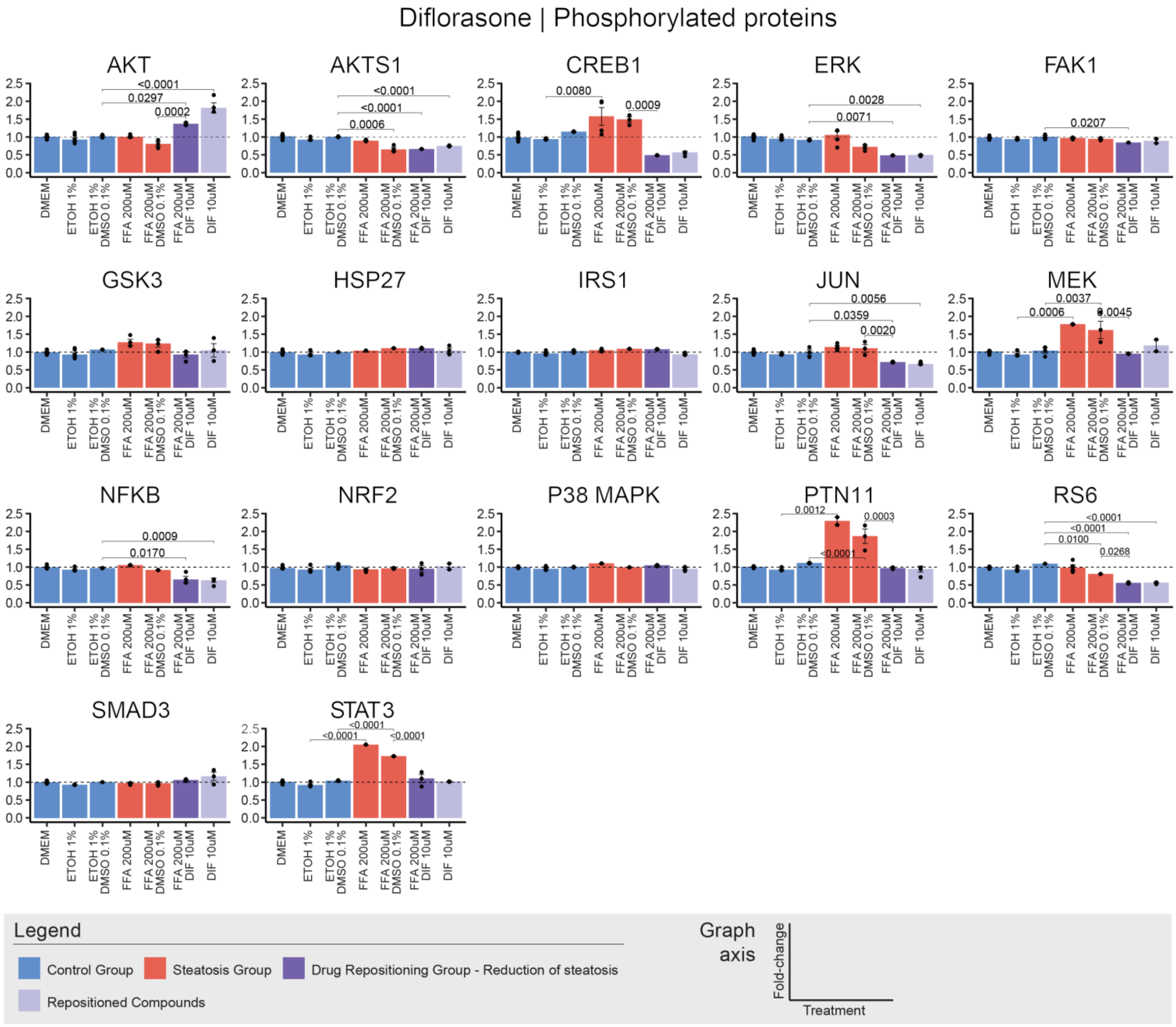

Figure S13 Statistical analysis of the effect of Diflorasone on the phosphorylated proteins measured. Related to Figure 5. *Bars represent the FC of median fluorescent intensity per protein in treated cells over respective controls. Data expressed as mean±SEM of at least n=3 independent experiments, and the p-value is denoted by brackets.*

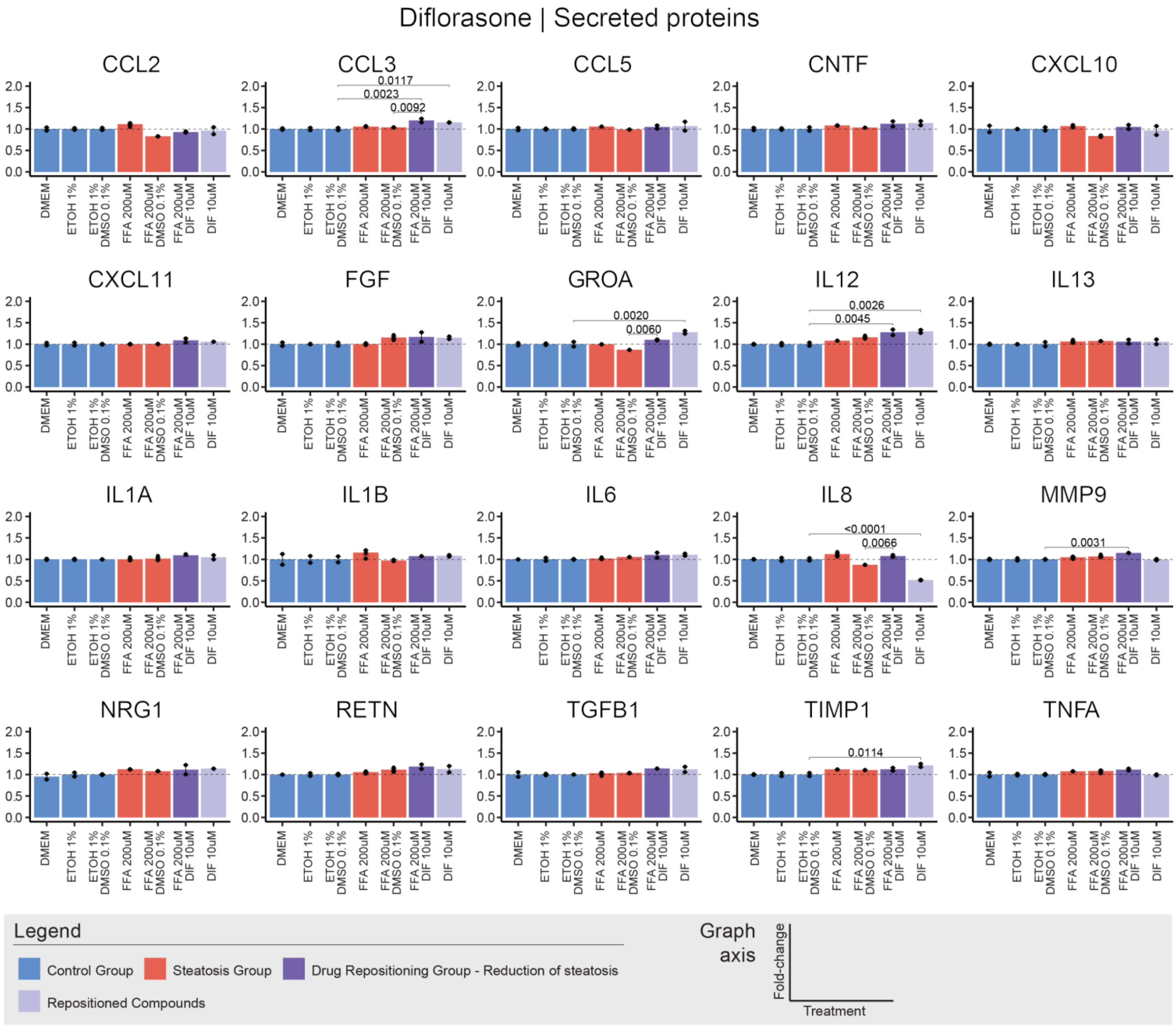

Figure S14 Statistical analysis of the effect of Diflorasone on the secreted proteins measured. Related to Figure 5. *Bars represent the FC of median fluorescent intensity per protein in treated cells over respective controls. Data expressed as mean±SEM of at least n=3 independent experiments, and the p-value is denoted by brackets.*

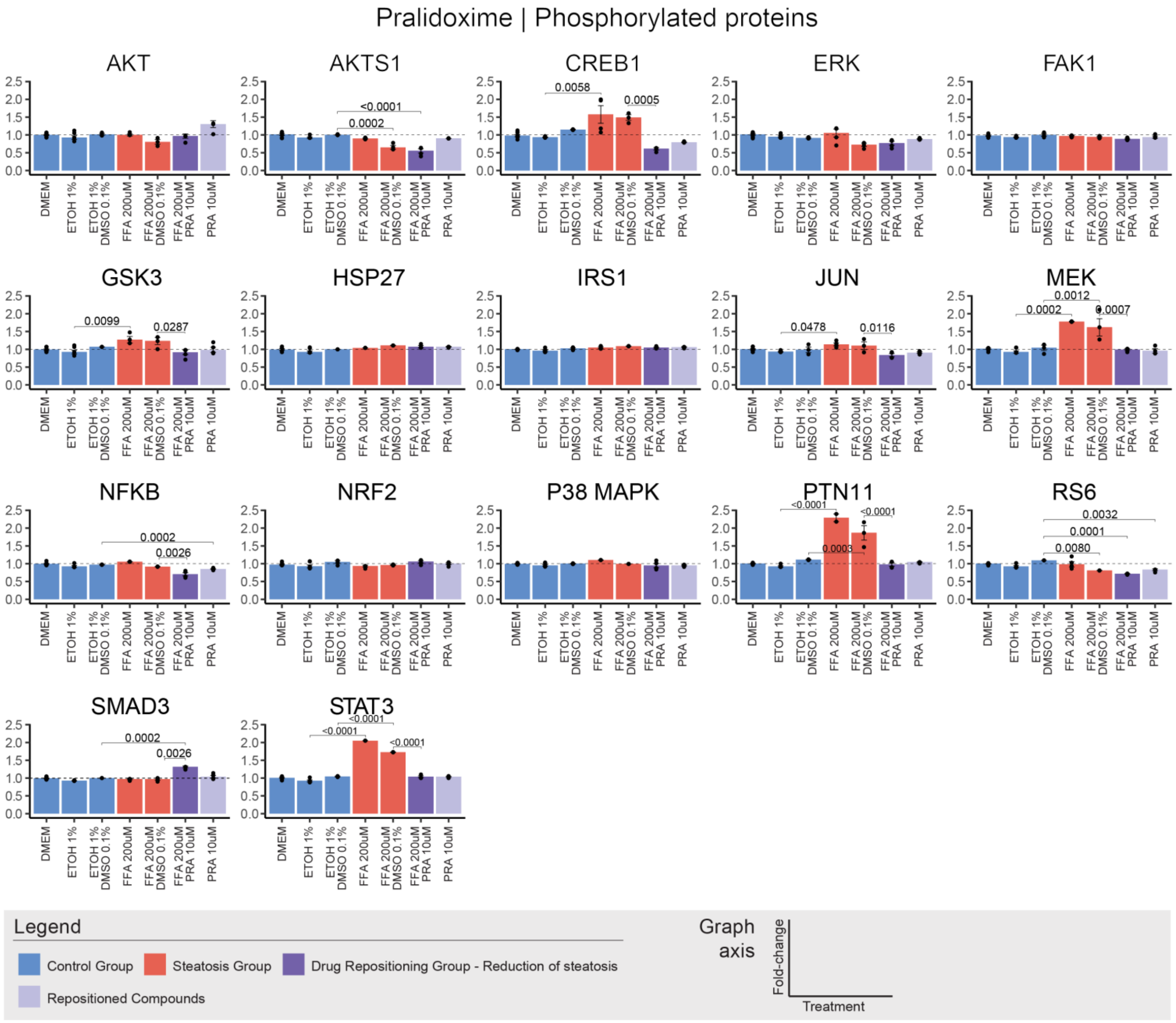

Figure S15 Statistical analysis of the effect of Pralidoxime on the phosphorylated proteins measured. Related to Figure 5. *Bars represent the FC of median fluorescent intensity per protein in treated cells over respective controls. Data expressed as mean±SEM of at least n=3 independent experiments, and the p-value is denoted by brackets.*

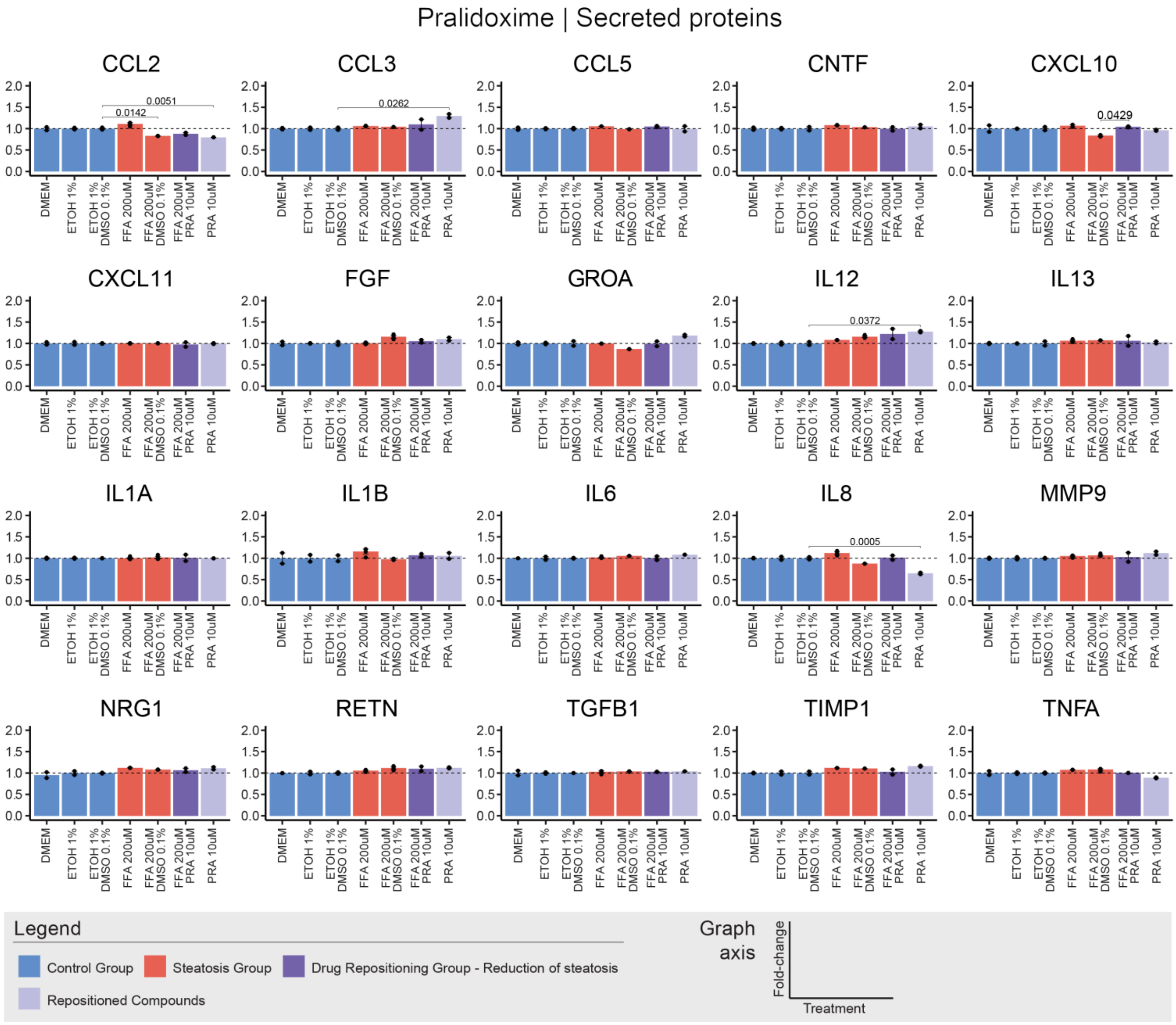

Figure S16 Statistical analysis of the effect of Pralidoxime on the secreted proteins measured. Related to Figure 5. *Bars represent the FC of median fluorescent intensity per protein in treated cells over respective controls. Data expressed as mean±SEM of at least n=3 independent experiments, and the p-value is denoted by brackets.*

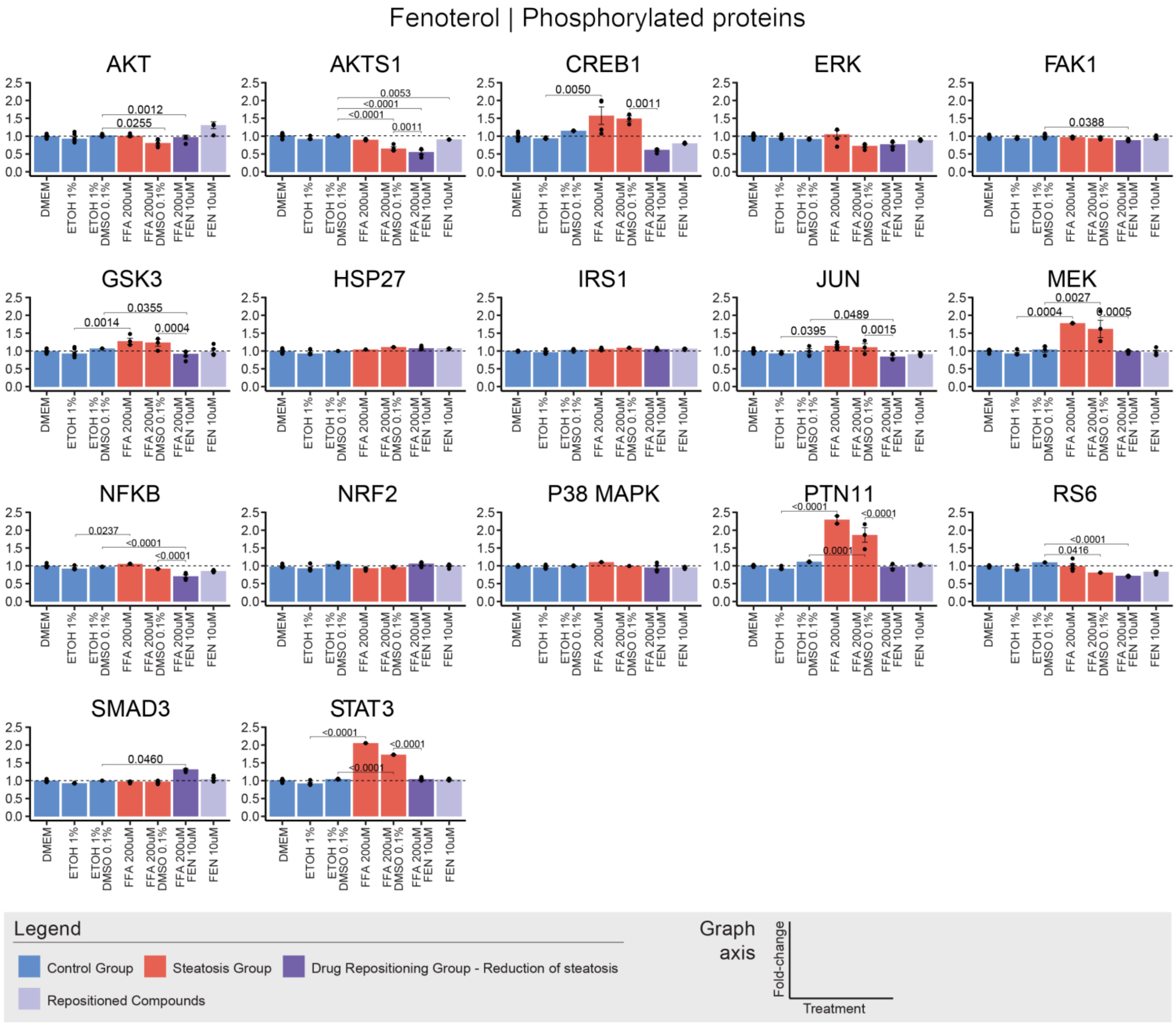

Figure S17 Statistical analysis of the effect of Fenoterol on the phosphorylated proteins measured. Related to Figure 5. *Bars represent the FC of median fluorescent intensity per protein in treated cells over respective controls. Data expressed as mean±SEM of at least n=3 independent experiments, and the p-value is denoted by brackets.*

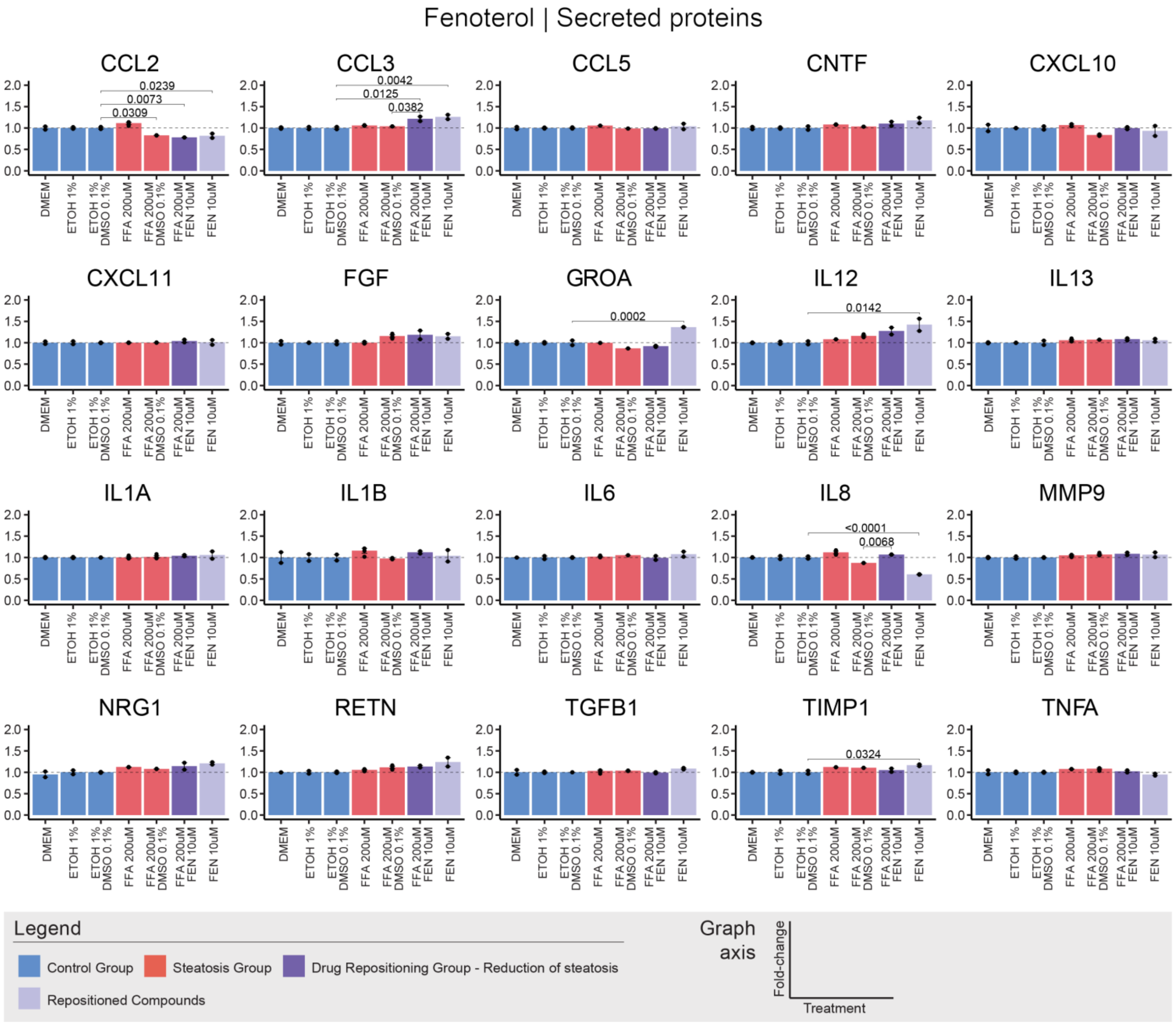

Figure S18 Statistical analysis of the effect of Fenoterol on the secreted proteins measured. Related to Figure 5. *Bars represent the FC of median fluorescent intensity per protein in treated cells over respective controls. Data expressed as mean±SEM of at least n=3 independent experiments, and the p-value is denoted by brackets.*

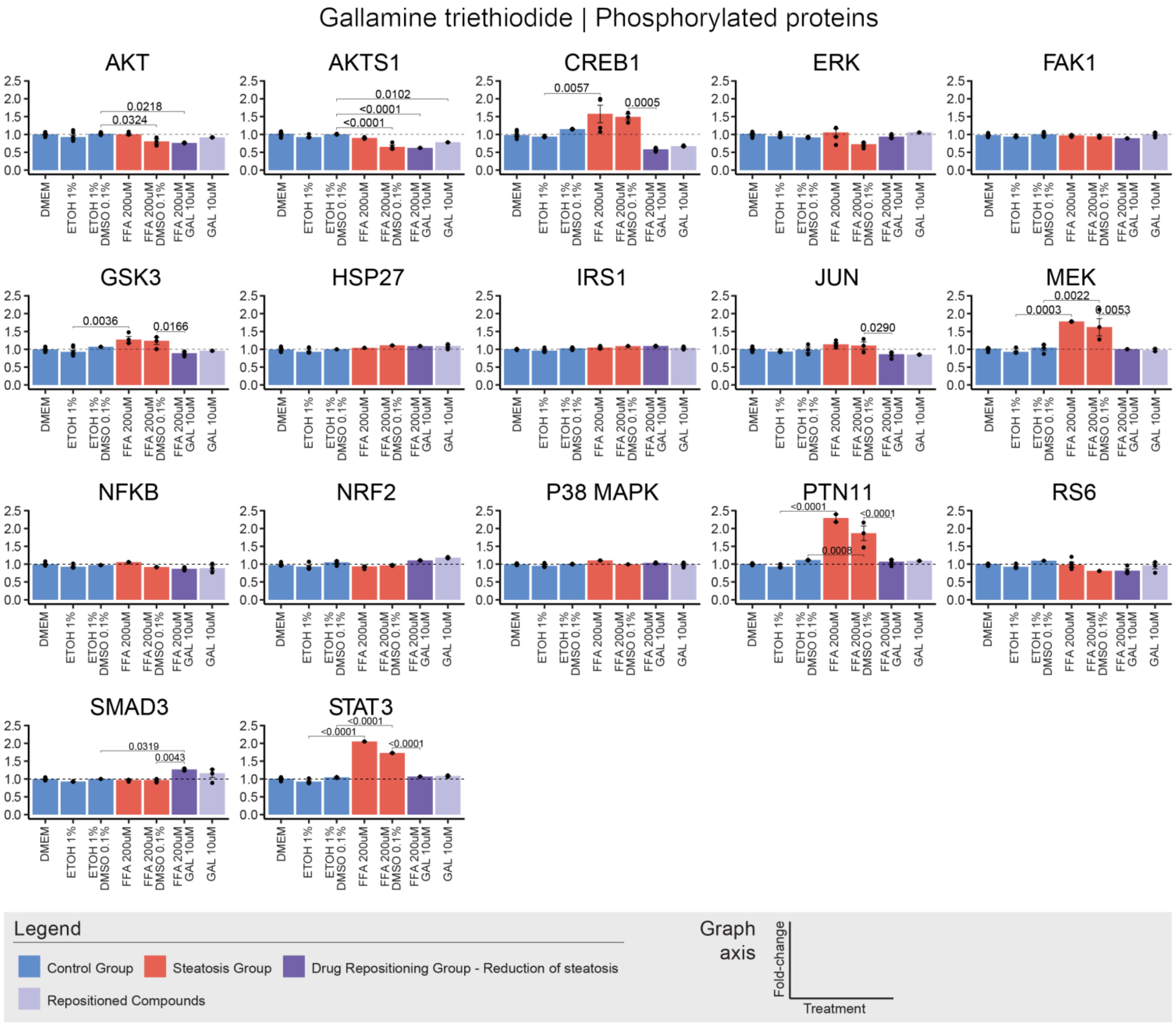

Figure S19 Statistical analysis of the effect of Gallamine triethiodide on the phosphorylated proteins measured. Related to Figure 5. *Bars represent the FC of median fluorescent intensity per protein in treated cells over respective controls. Data expressed as mean±SEM of at least n=3 independent experiments, and the p-value is denoted by brackets.*

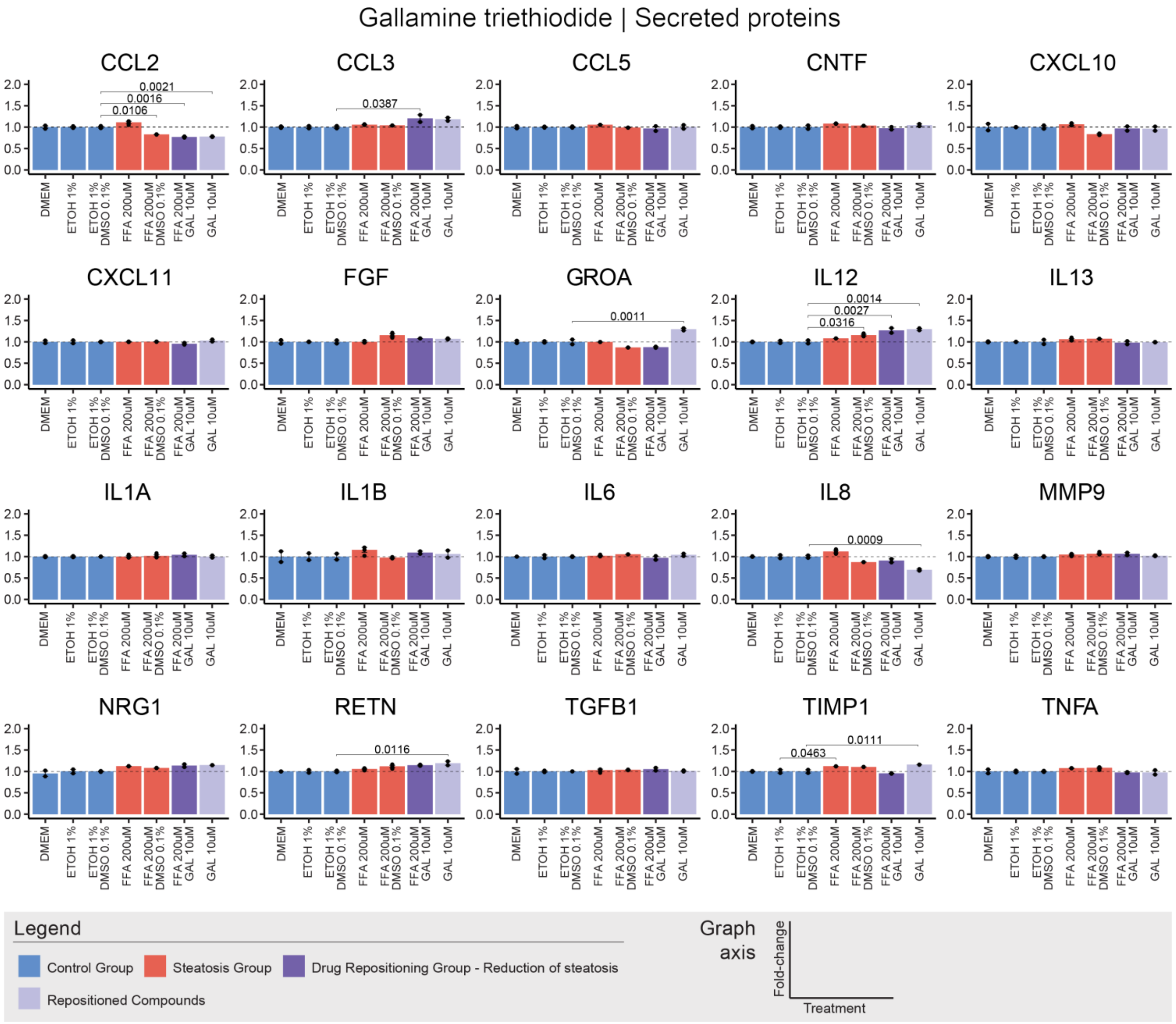

Figure S20 Statistical analysis of the effect of Gallamine triethiodide on the secreted proteins measured. Related to Figure 5. *Bars represent the FC of median fluorescent intensity per protein in treated cells over respective controls. Data expressed as mean±SEM of at least n=3 independent experiments, and the p-value is denoted by brackets.*
